# Supplementary material for: New Alkylpyridinium Anthraquinone, Isocoumarin, C-Glucosyl Resorcinol Derivative and Prenylated Pyranoxanthones from the Culture of a Marine Sponge-Associated Fungus, Aspergillus stellatus KUFA 2017
Source: Mar Drugs. 2022 Oct 27;20(11):672. doi: 10.3390/md20110672 (PMC9696483; doi:10.3390/md20110672)
Supplement: Supplementary file 1 [file marinedrugs-20-00672-s001.zip › marinedrugs-1957955-supplementary.pdf]

Supplementary Materials

New Alkylpyridinium Anthraquinone, Isocoumarin, C-Glucosyl Resorcinol Derivative and Prenylated Pyranoxanthones from the Culture of a Marine Sponge-Associated Fungus, *Aspergillus stellatus* KUFA 2017

Fátima P. Machado [1,2], Inês C. Rodrigues [1], Luís Gales [1,3], José A. Pereira [1,2], Paulo M. Costa [1,2], Tida Dethoup [4], Sharad Mistry [5], Artur M. S. Silva [6], Vitor Vasconcelos [2,7] and Anake Kijjoa [1,2,*]

1 ICBAS-Instituto de Ciências Biomédicas Abel Salazar, Rua de Jorge Viterbo Ferreira, 228, 4050-313 Porto, Portugal
2 Interdisciplinary Centre of Marine and Environmental Research (CIIMAR), Terminal de Cruzeiros do Porto de Leixões, Av. General Norton de Matos s/n, 4450-208 Matosinhos, Portugal
3 Instituto de Biologia Molecular e Celular (i3S-IBMC), Universidade do Porto, Rua de Jorge Viterbo Ferreira, 228, 4050-313 Porto, Portugal
4 Department of Plant Pathology, Faculty of Agriculture, Kasetsart University, Bangkok 10240, Thailand
5 Department of Chemistry, University of Leicester, University Road, Leicester LE 7 RH, UK
6 Departamento de Química & QOPNA, Universidade de Aveiro, 3810-193 Aveiro, Portugal
7 FCUP-Faculty of Sciences, University of Porto, Rua do Campo Alegre, s/n, 4169-007 Porto, Portugal
* Correspondence: ankijjoa@icbas.up.pt; Tel.: +351-962712474; Fax: +351-22-206-2232

Figure S1. ^{1}H NMR spectrum of **1** ($CDCl_3$, 300MHz).

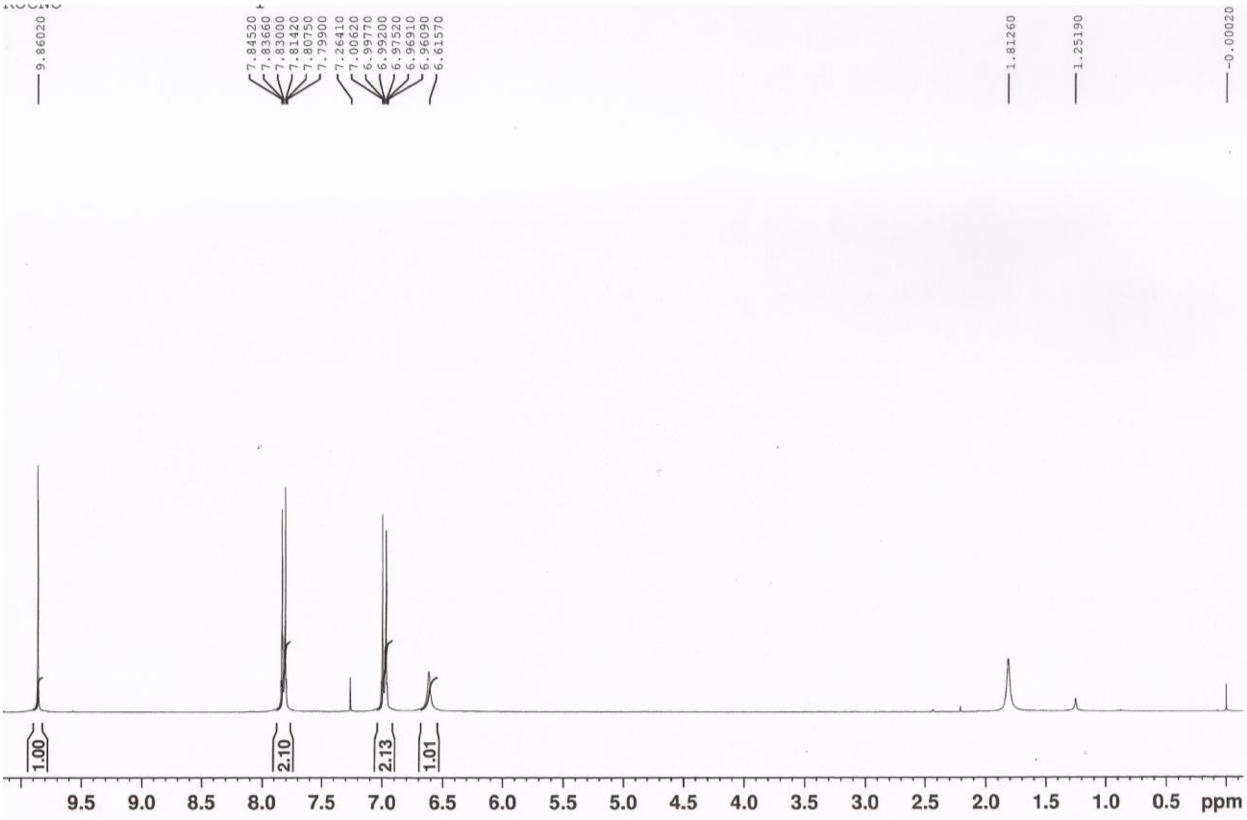

Figure S2. ^{13}C NMR spectrum of **1** ($CDCl_3$, 75 MHz).

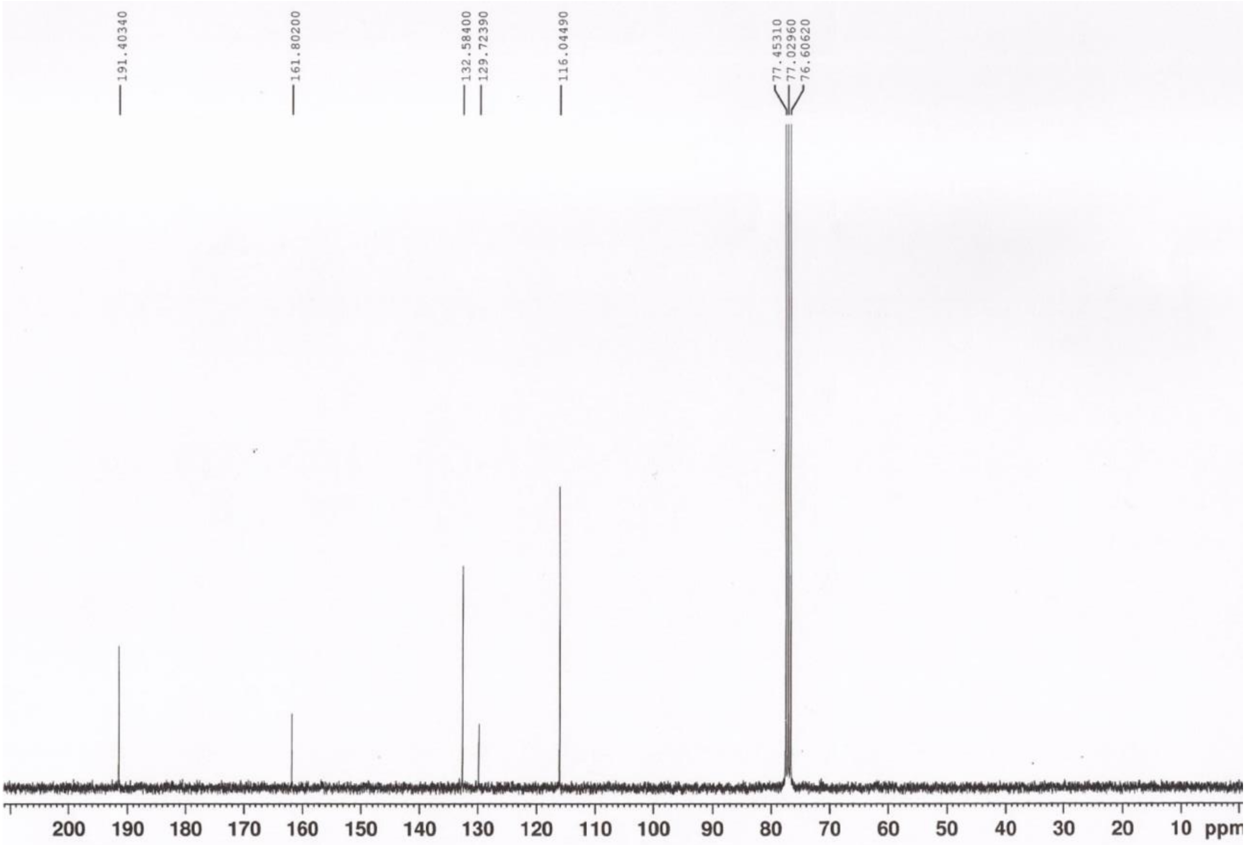

Figure S3. ^{1}H NMR spectrum of **2** ($CDCl_3$, 300MHz).

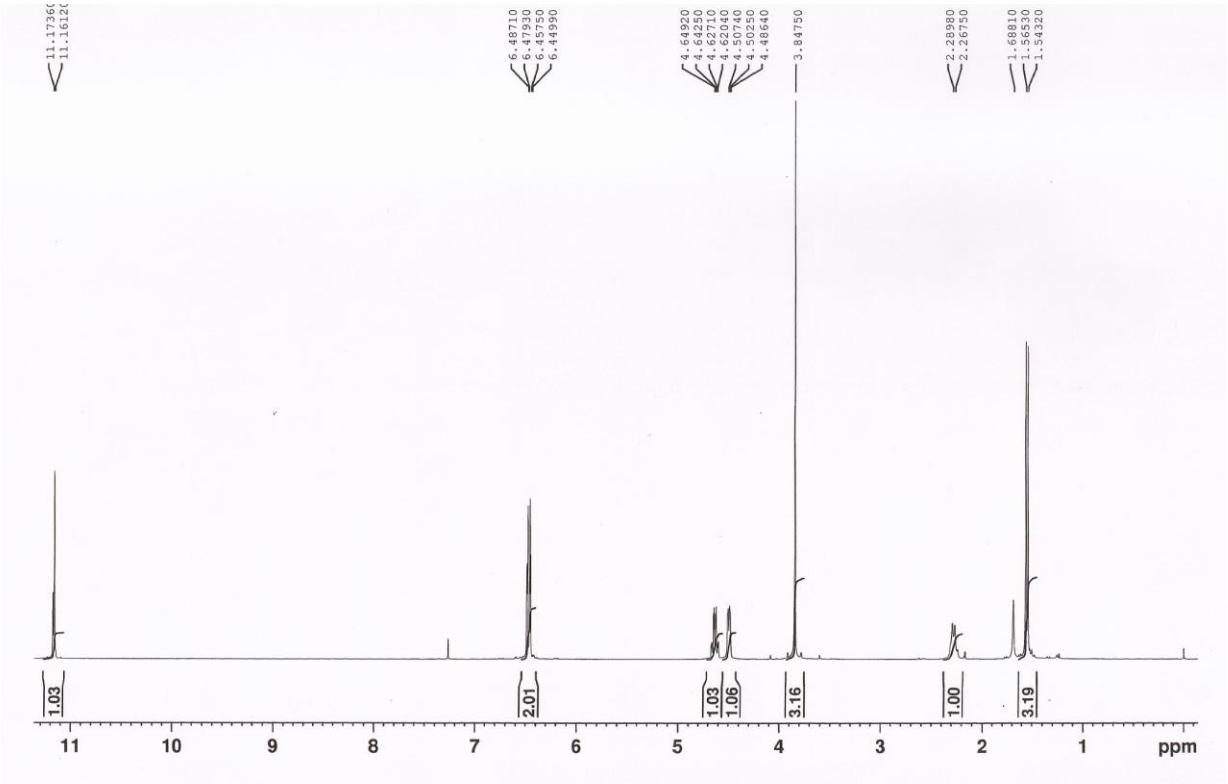

Figure S4. ^{13}C NMR spectrum of **2** ($CDCl_3$, 75 MHz).

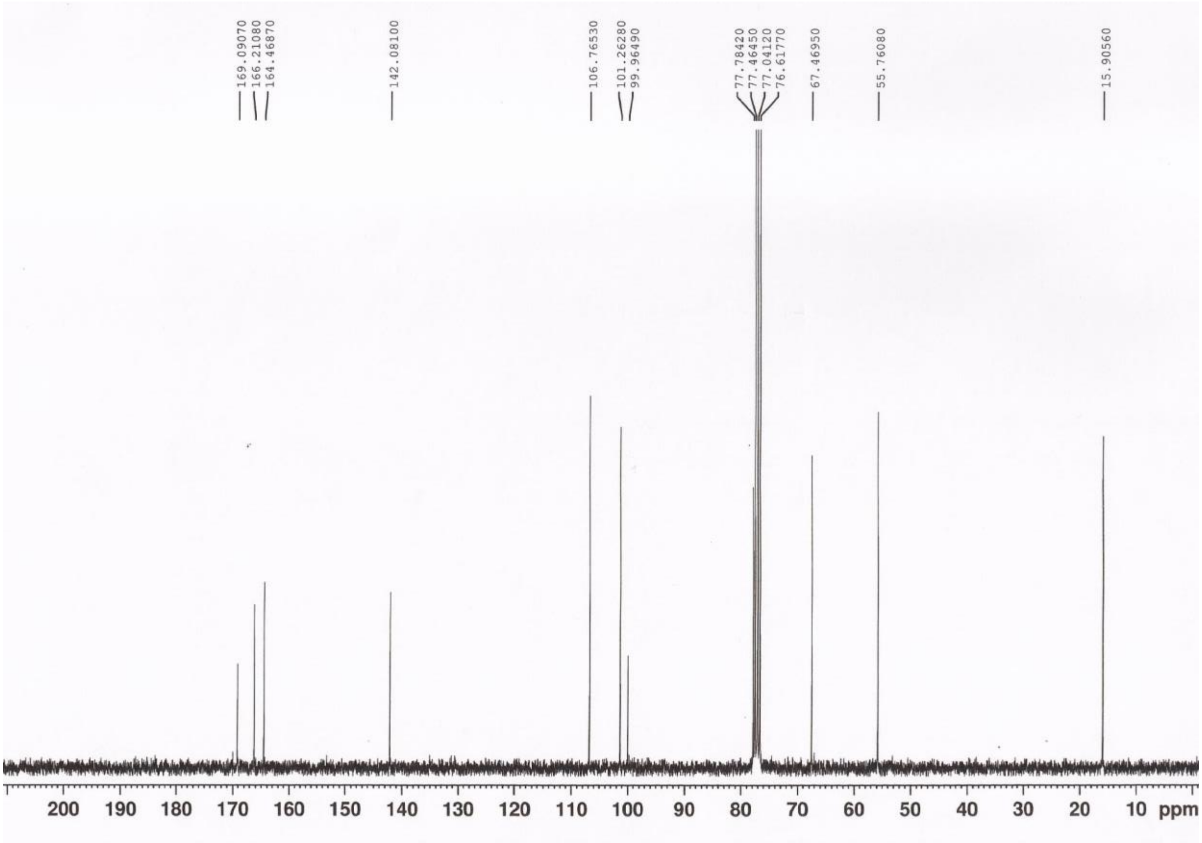

Figure S5. COSY spectrum of **2** ($CDCl_3$, 300 MHz).

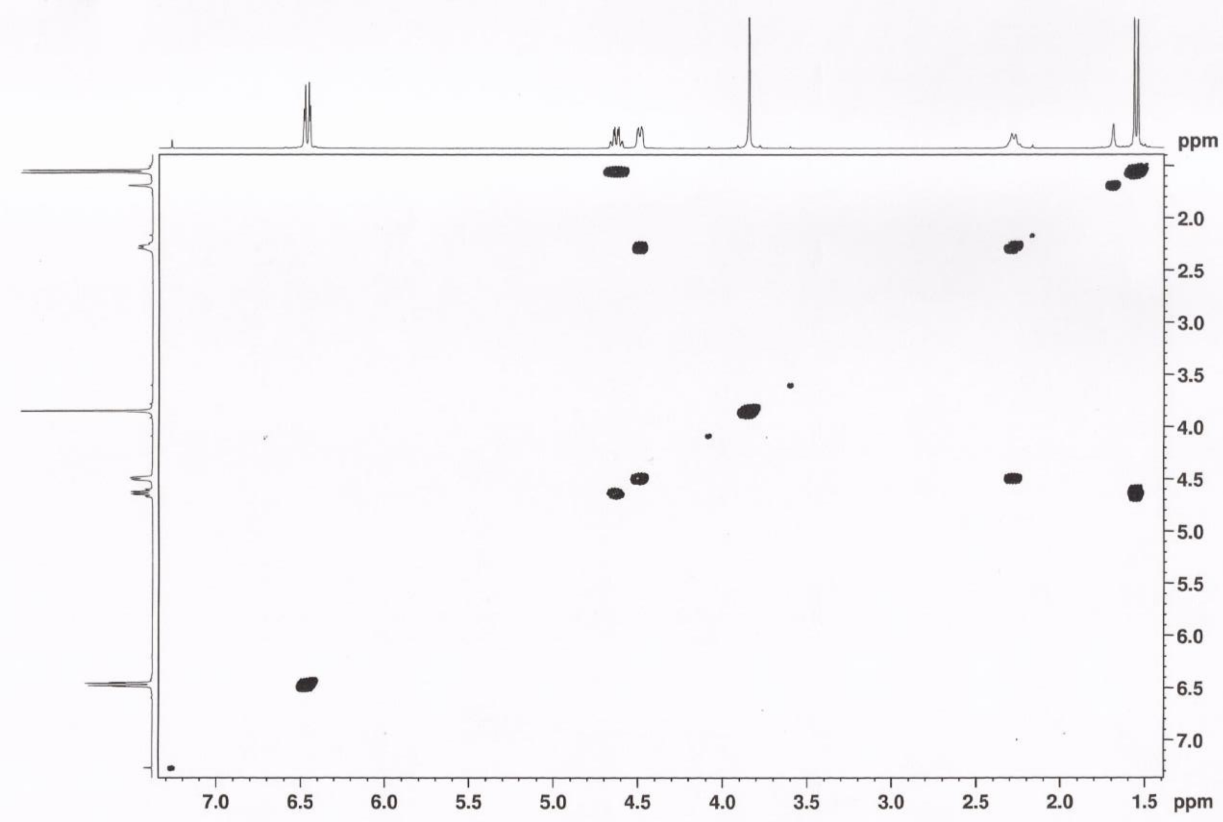

Figure S6. HSQC spectrum of **2** ($CDCl_3$, 300 MHz).

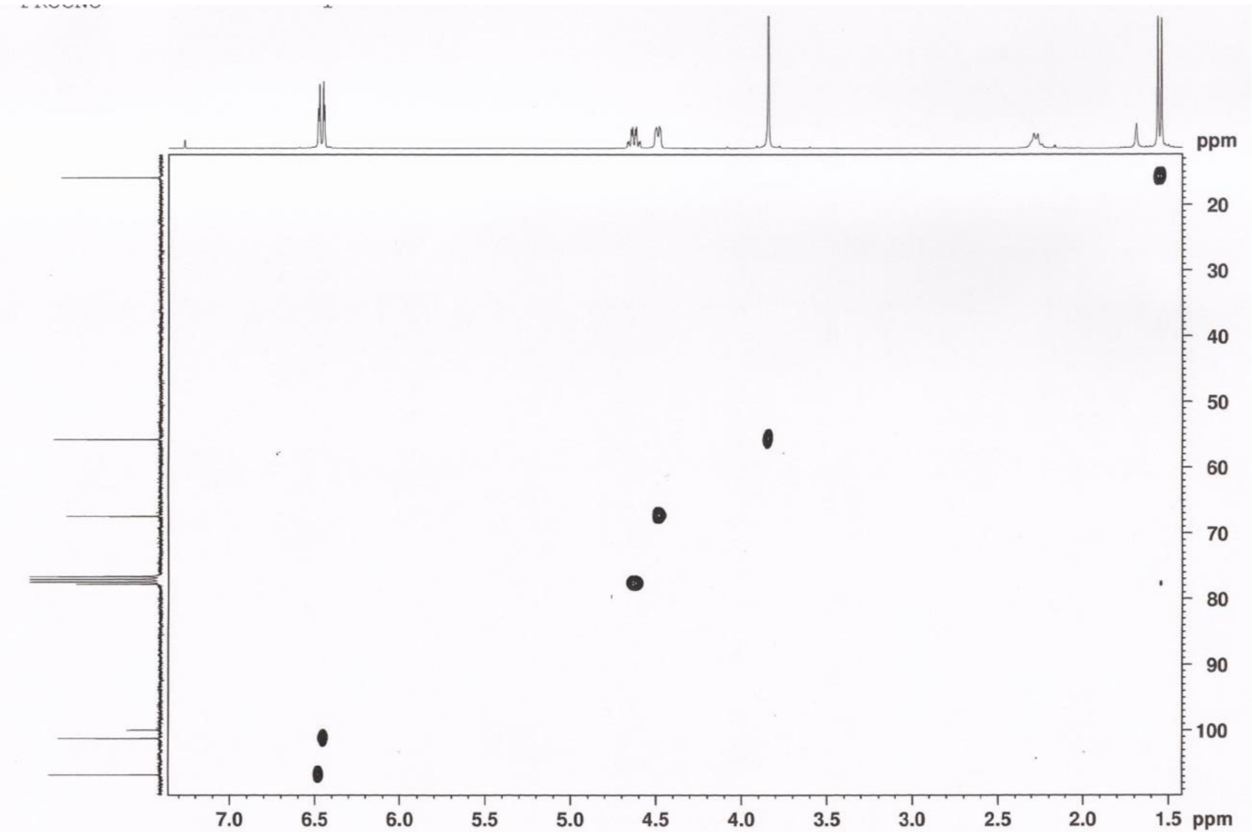

Figure S7. HMBC spectrum of **2** ($CDCl_3$, 300 MHz).

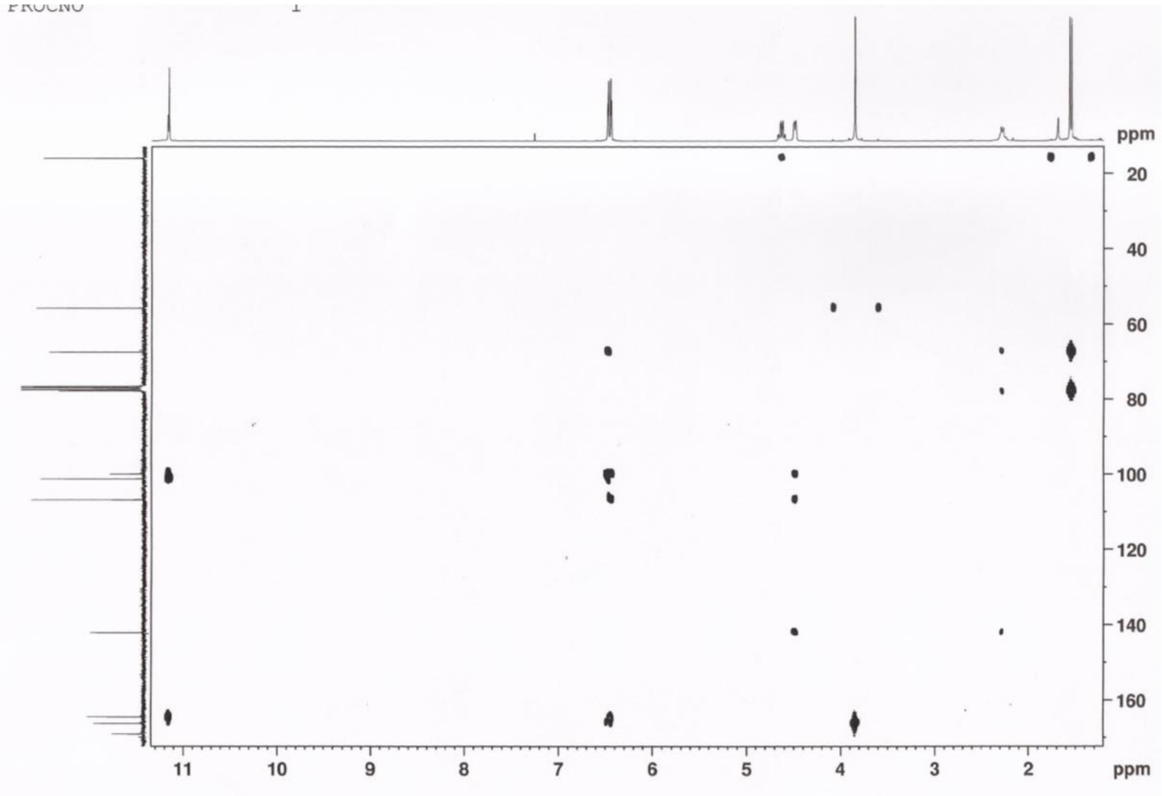

Figure S8. NOESY spectrum of **2** ($CDCl_3$, 300 MHz).

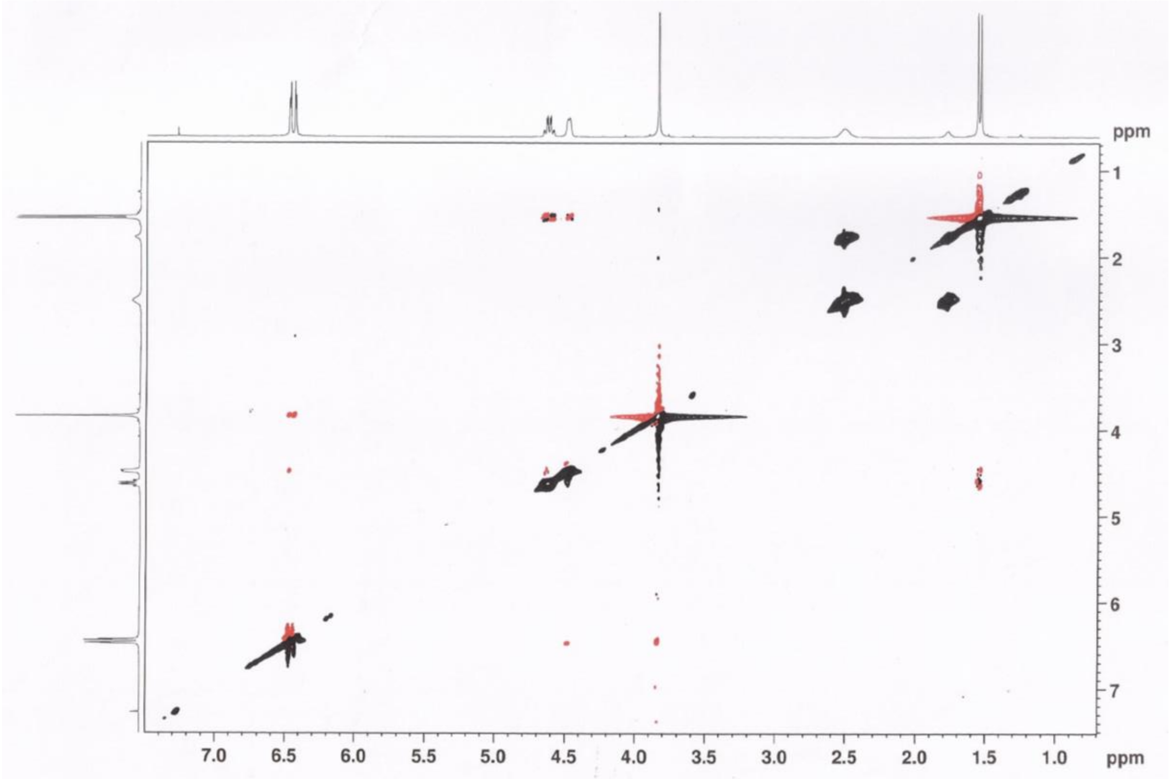

Figure S9. ^{1}H NMR spectrum of **3a** ($CDCl_3$, 300 MHz)

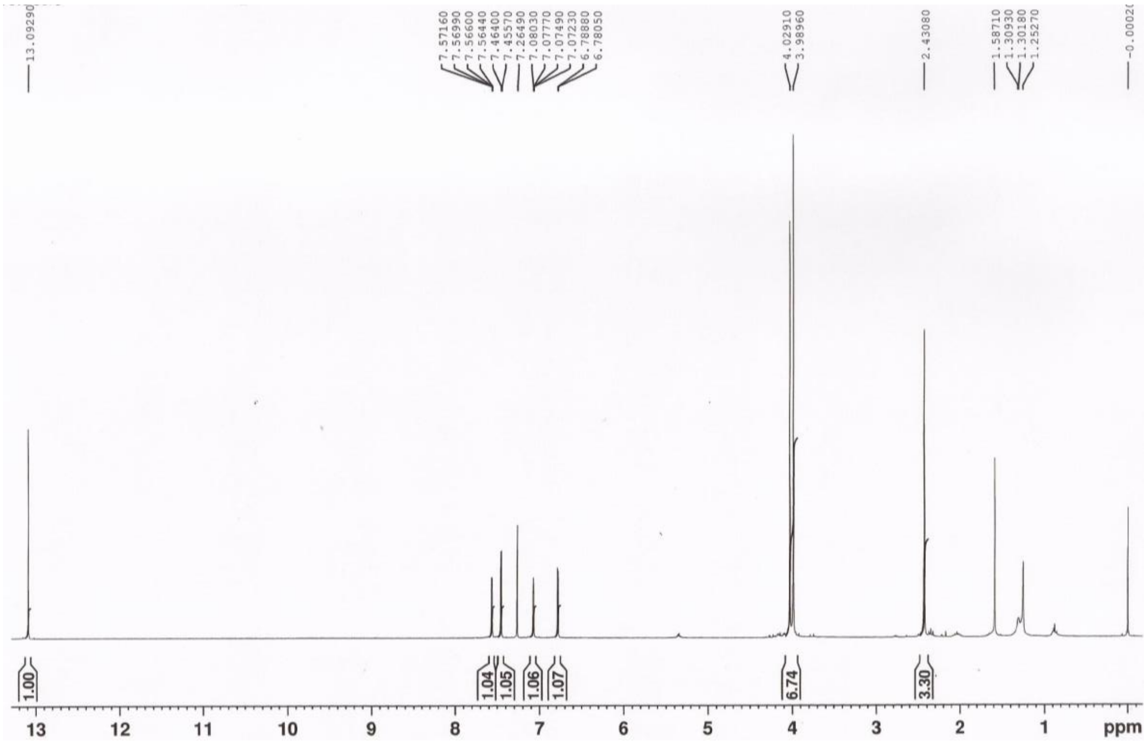

Figure S10. ^{13}C NMR spectrum of **3a** ($CDCl_3$, 75 MHz)

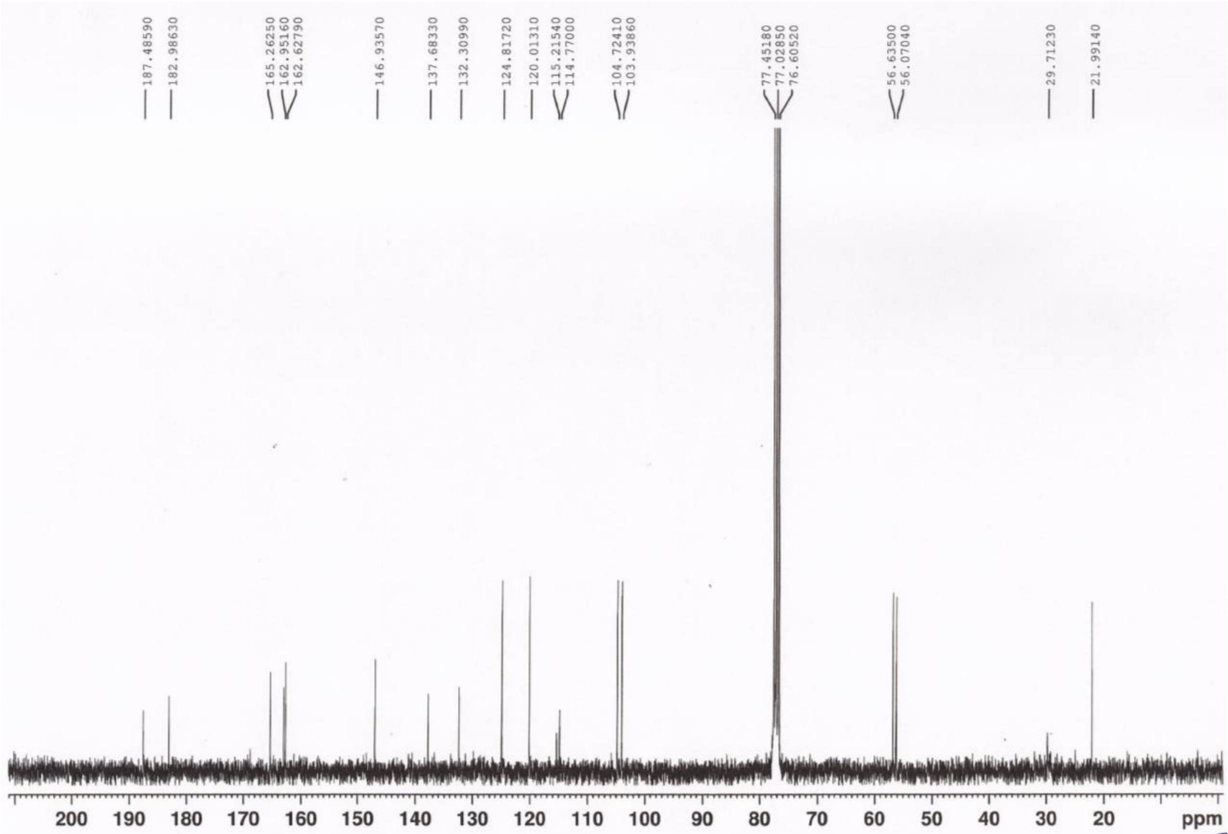

Figure S 11. COSY spectrum of **3a** ($CDCl_3$, 300 MHz)

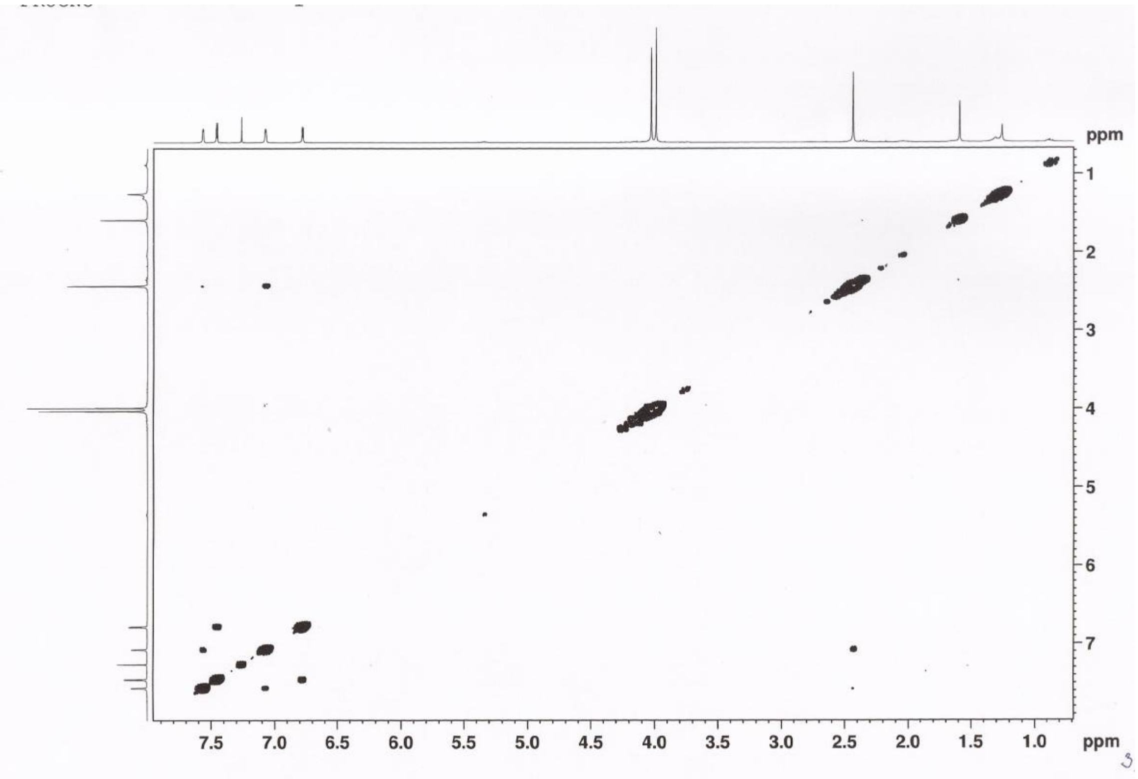

Figure S12. HSQC spectrum of **3a** ($CDCl_3$, 300 MHz)

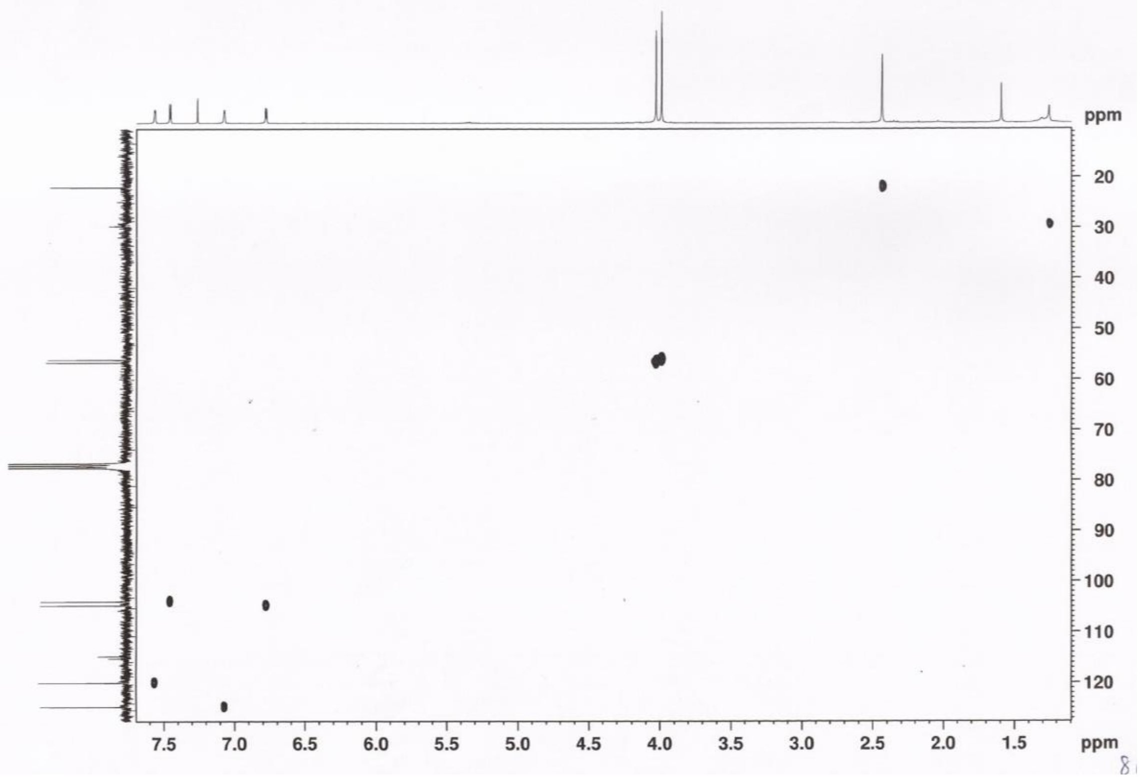

Figure S13. HMBC spectrum of **3a** ($CDCl_3$, 300 MHz)

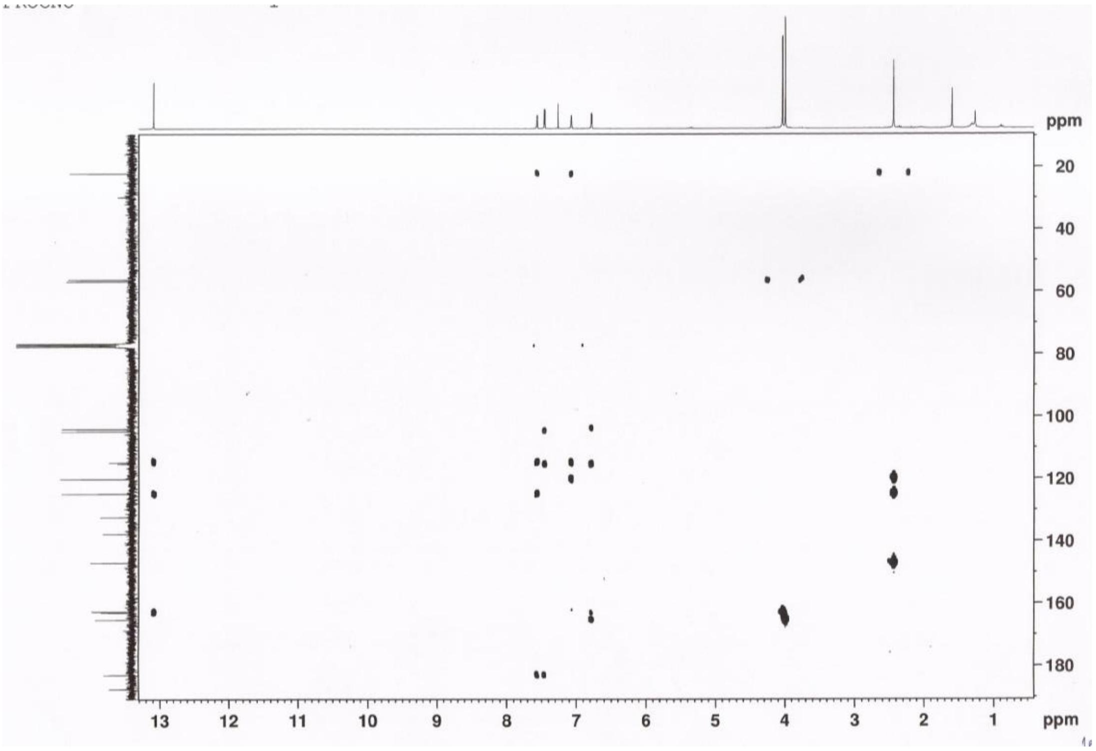

Figure S14. ^{1}H NMR spectrum of **3b** ($CDCl_3$, 300 MHz)

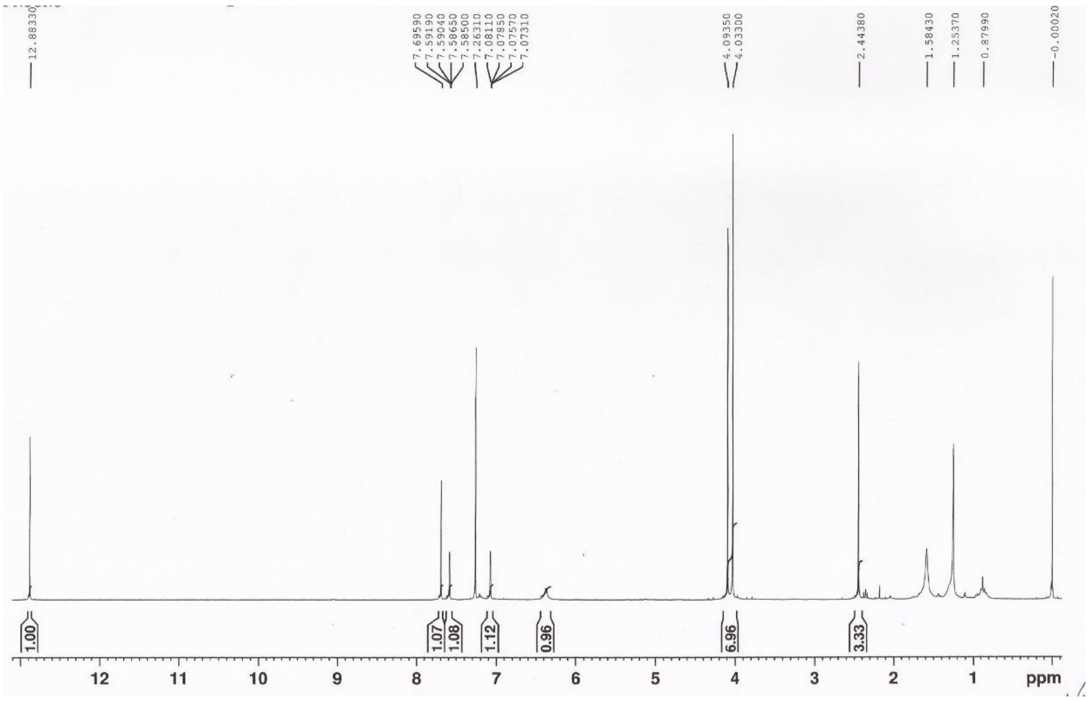

Figure S15. ^{13}C NMR spectrum of **3b** ($CDCl_3$, 75 MHz)

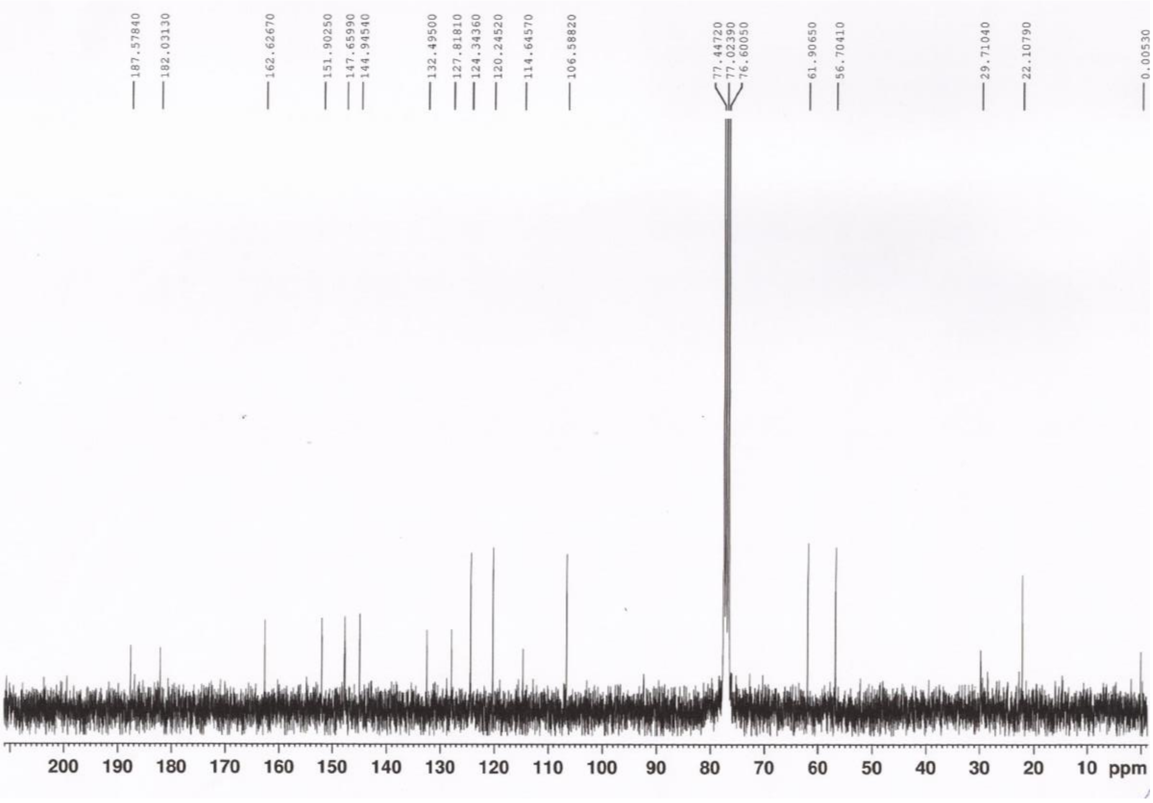

Figure S16. COSY spectrum of **3b** ($CDCl_3$, 300 MHz)

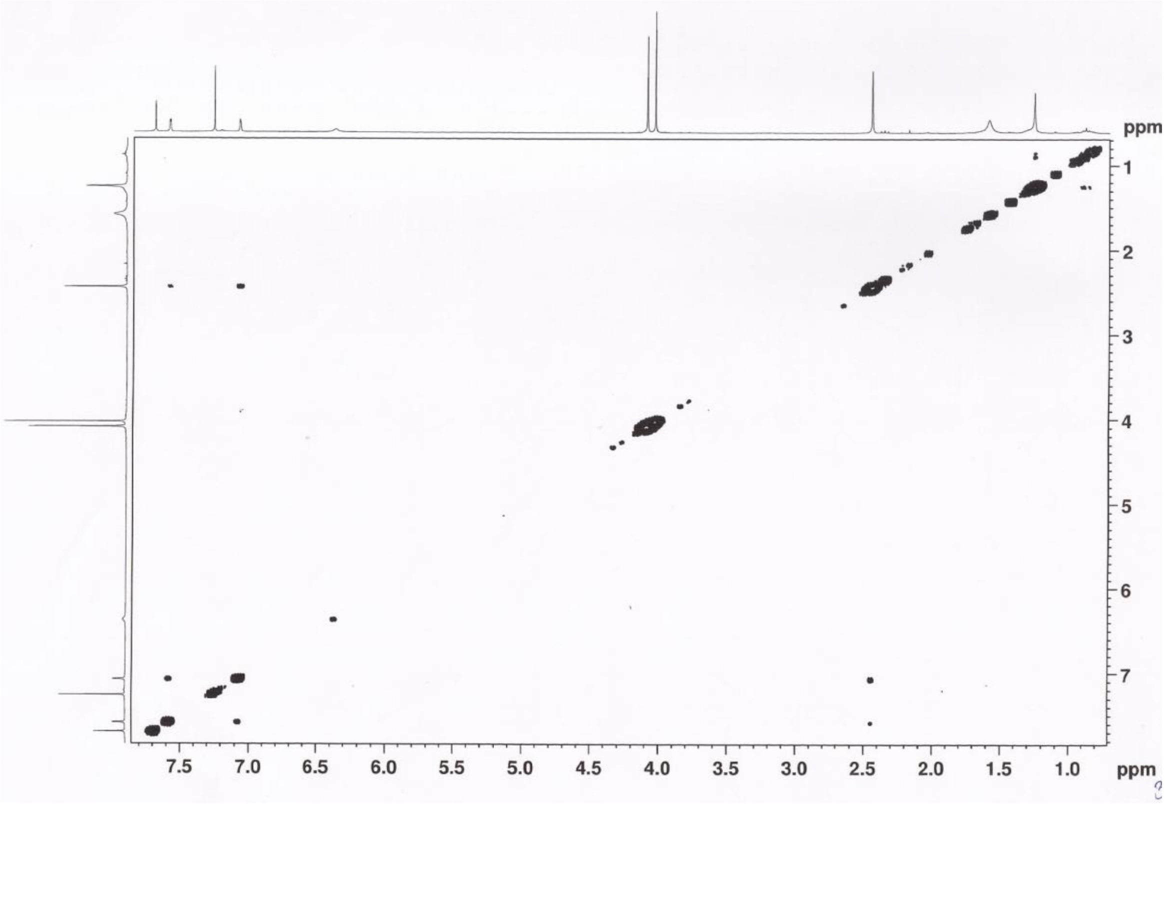

Figure S17. HSQC spectrum of **3b** ($CDCl_3$, 300 MHz).

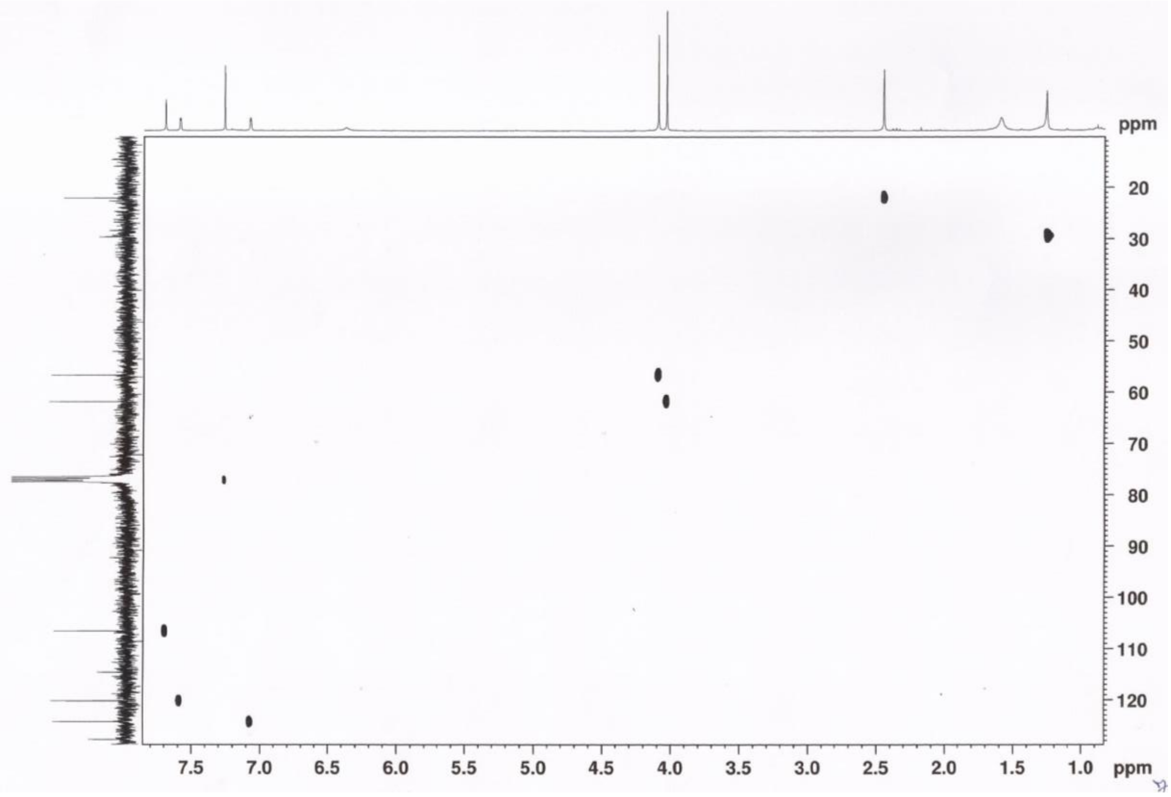

Figure S18. HMBC spectrum of **3b** ($CDCl_3$, 300 MHz)

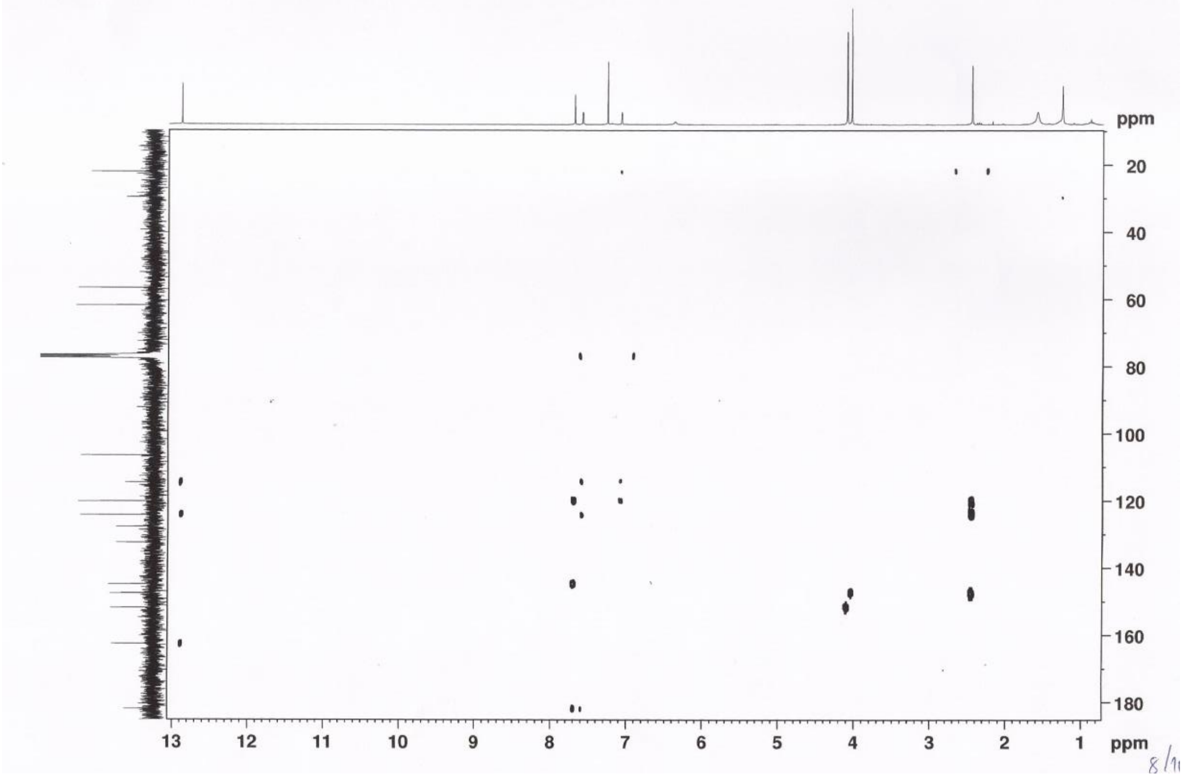

Figure S19. ^{1}H NMR spectrum of **3c** ($CDCl_3$, 300 MHz).

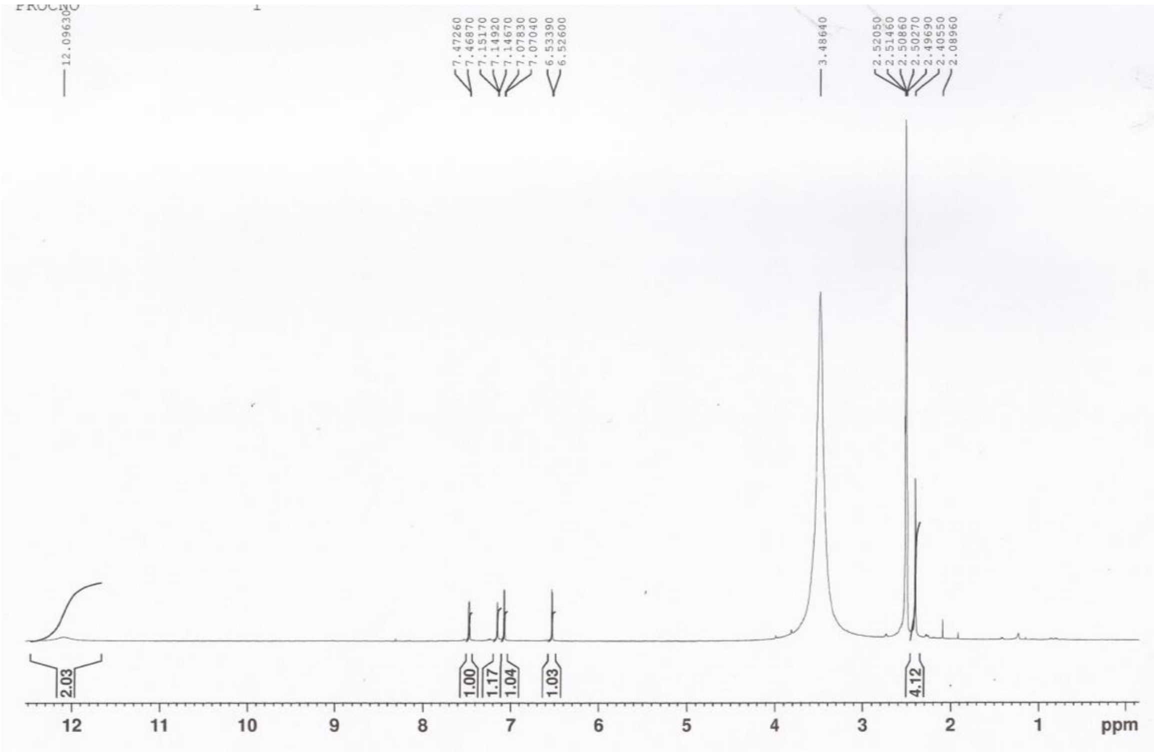

Figure S20. ^{13}C NMR spectrum of **3c** ($CDCl_3$, 75 MHz).

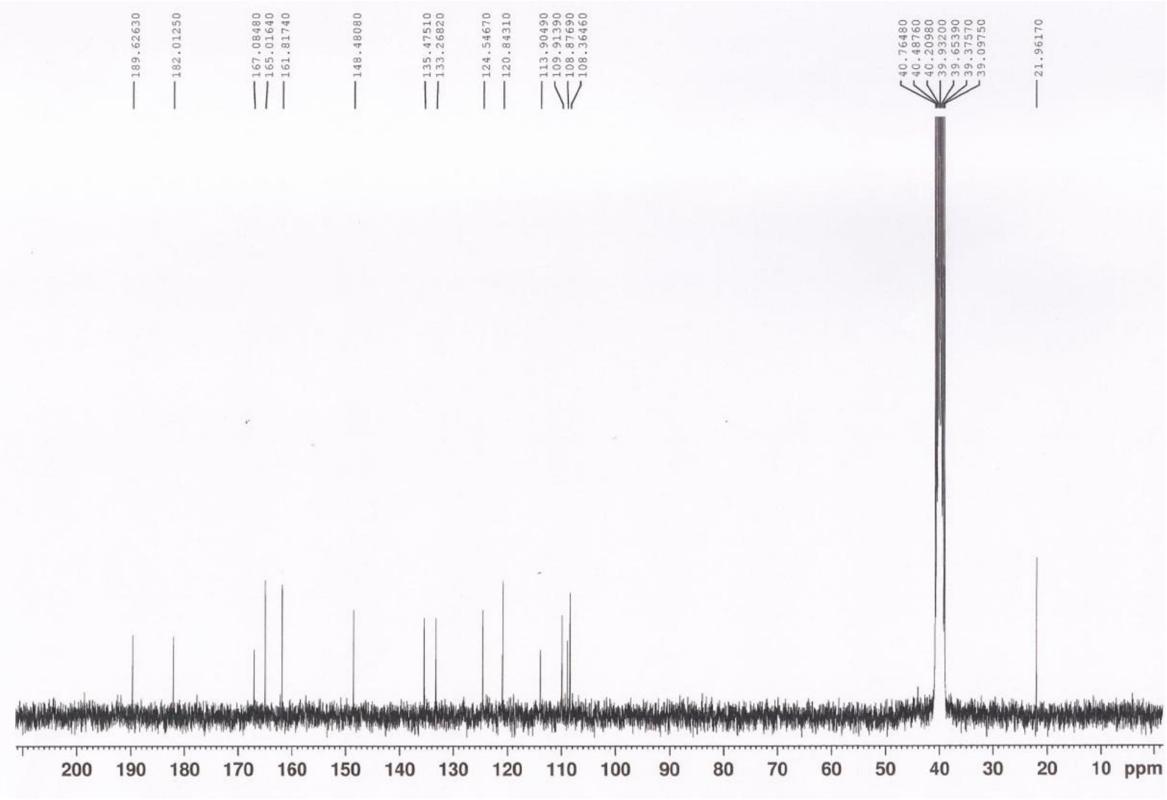

Figure S21. COSY spectrum of **3c** ($CDCl_3$, 300 MHz).

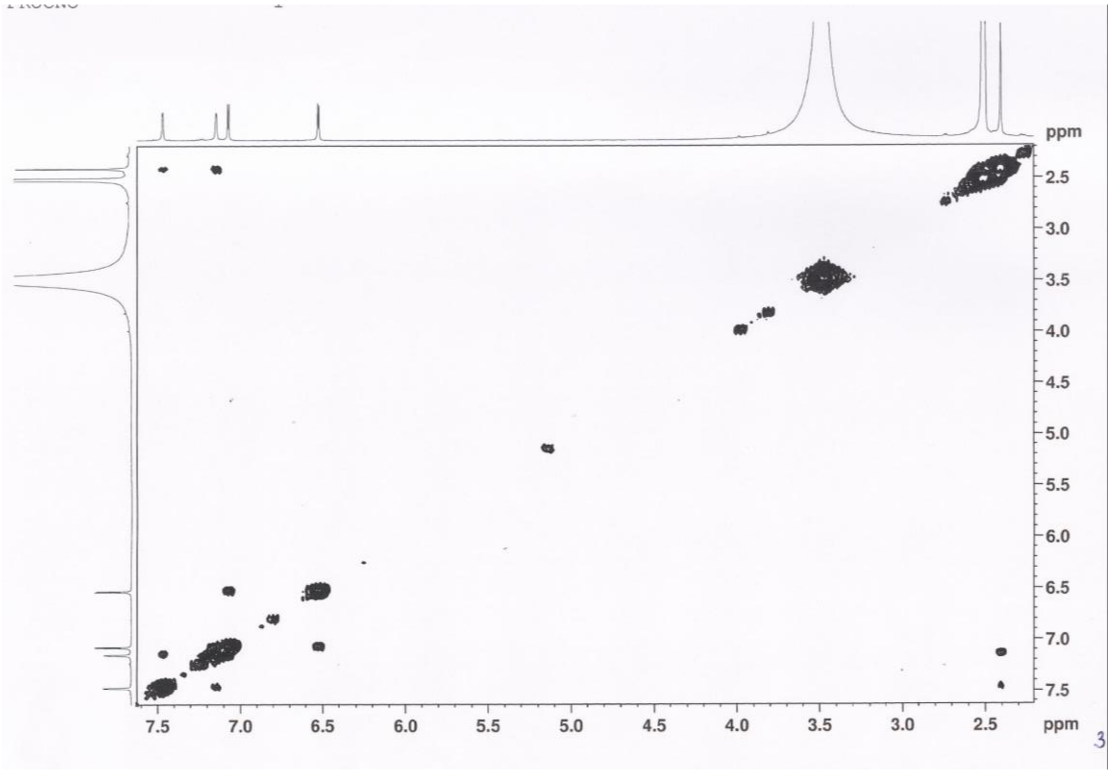

Figure S22. HSQC spectrum of **3c** ($CDCl_3$, 300 MHz).

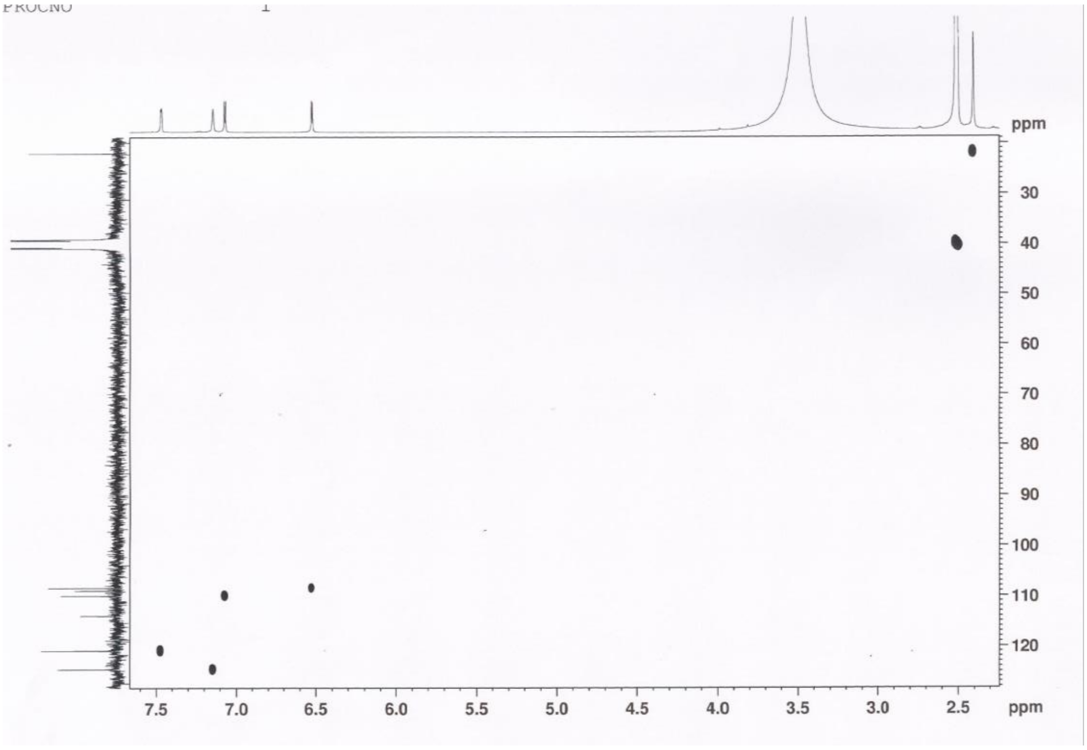

Figure S23. HMBC spectrum of **3c** ($CDCl_3$, 300 MHz).

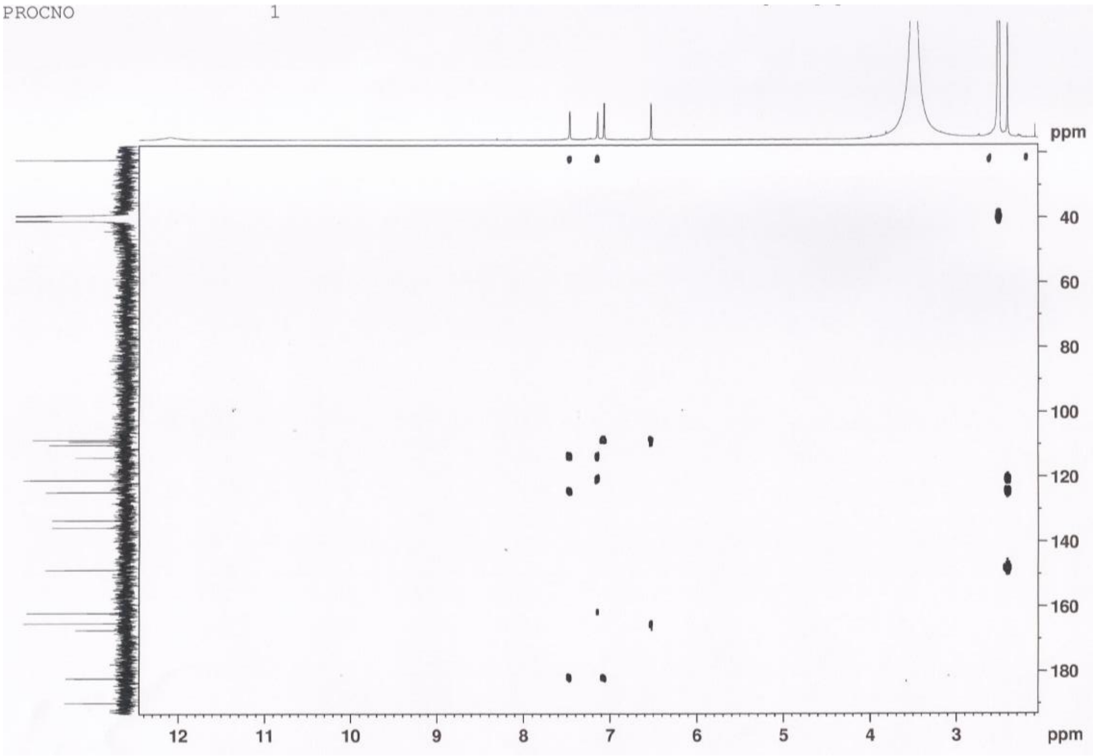

Figure S24. ^{1}H NMR spectrum of **4** (DMSO-d_6, 500 MHz).

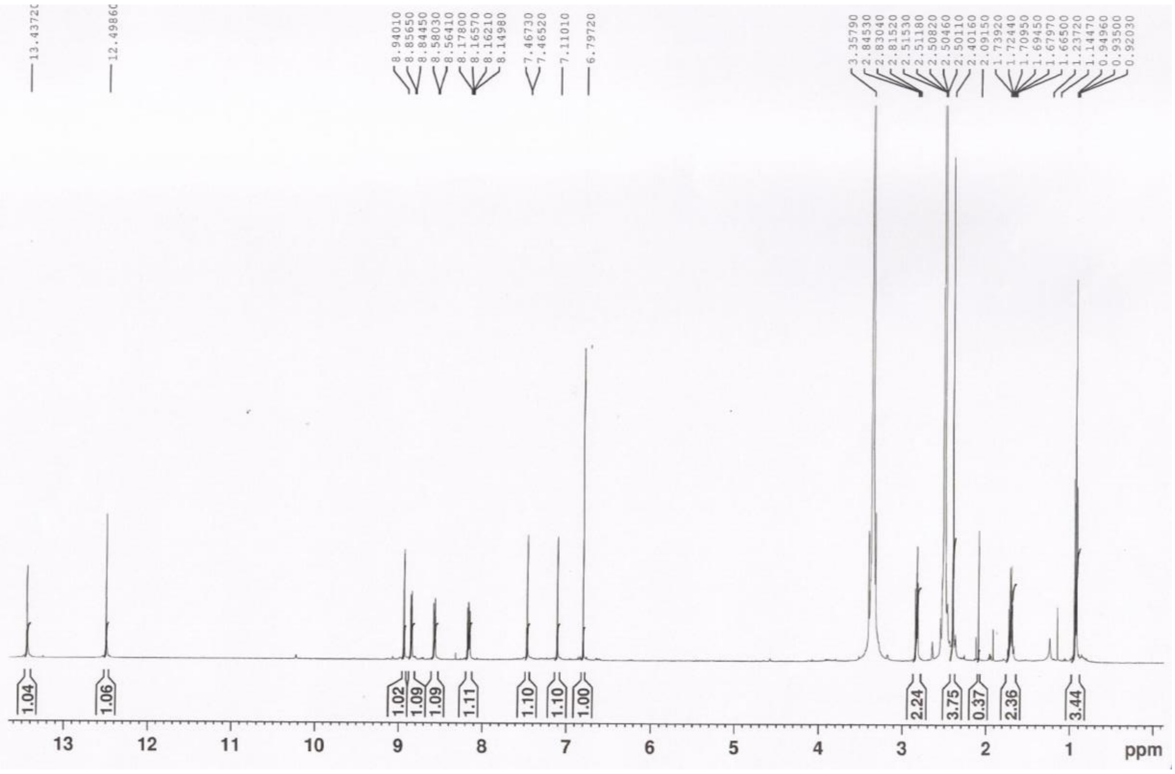

Figure S25. ^{13}C NMR spectrum of **4** (DMSO-*d*$_6$, 125 MHz).

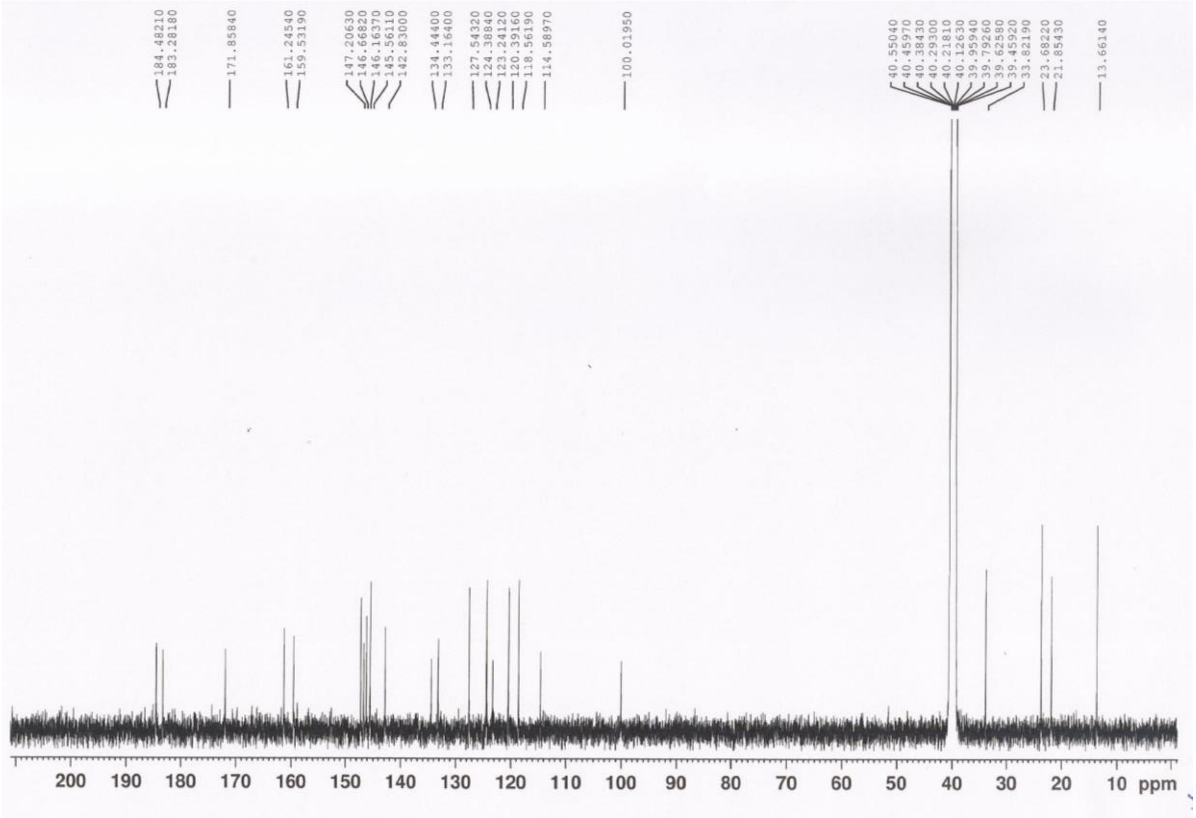

Figure S26. COSY spectrum of **4** (CD DMSO-*d*$_6$, 500 MHz).

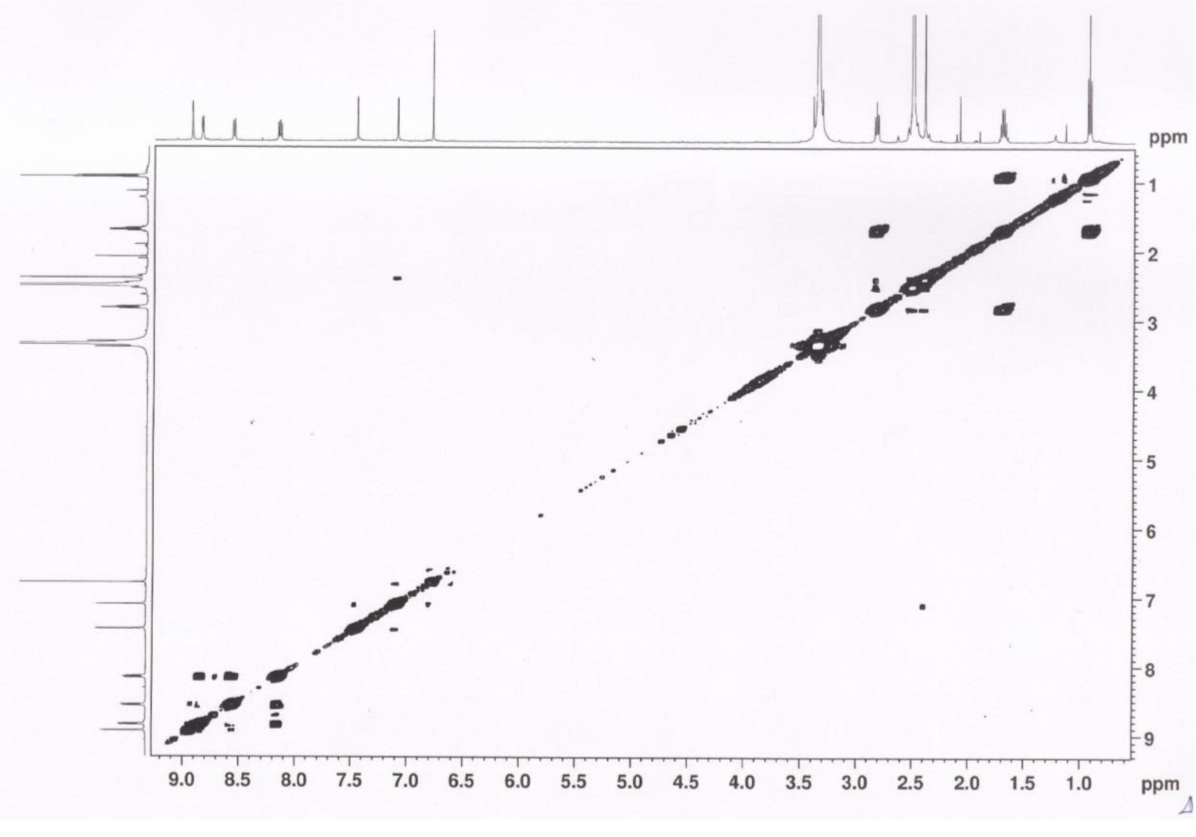

Figure S27. HSQC spectrum of **4** (DMSO-d_6, 500 MHz).

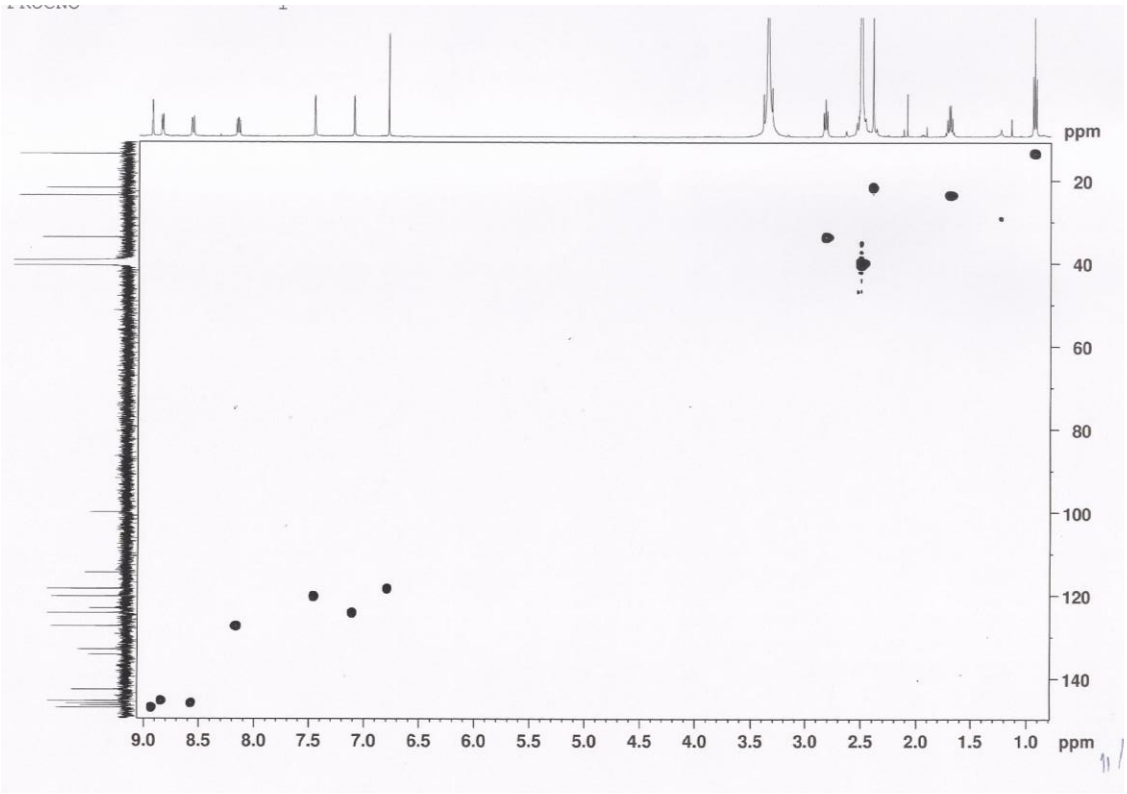

Figure S28. HMBC spectrum of **4** (DMSO-d_6, 500 MHz).

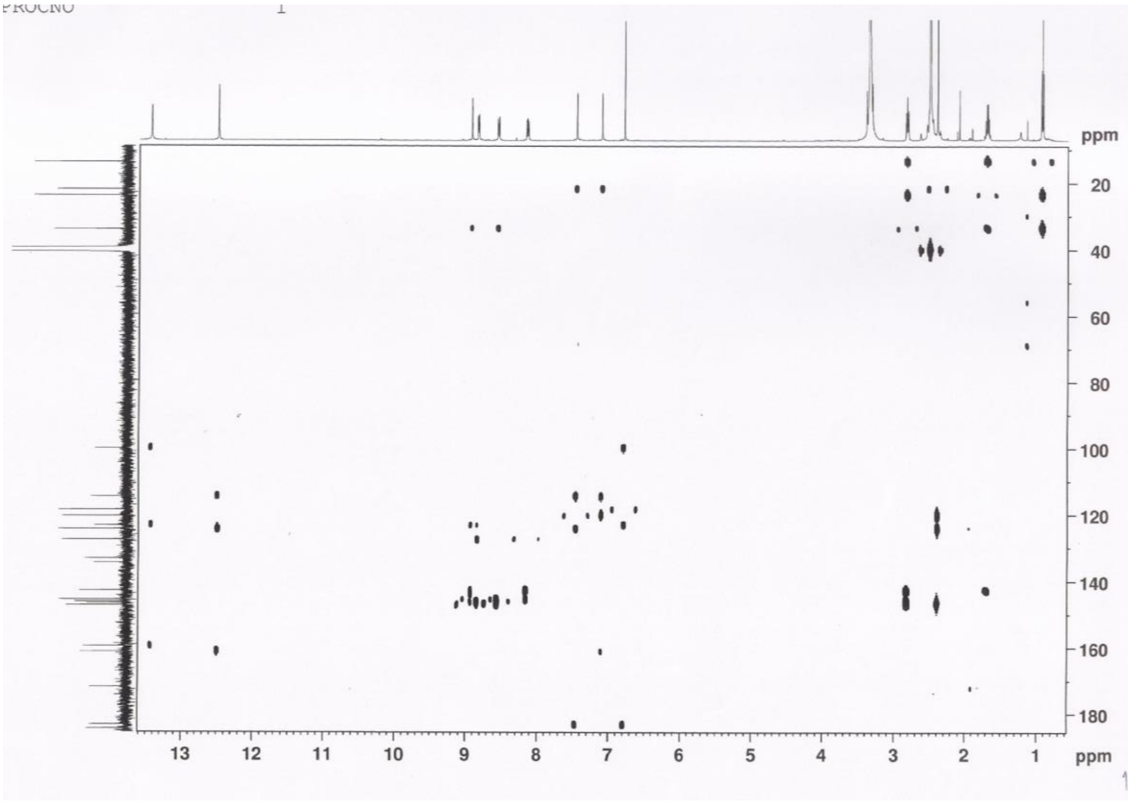

Figure S29. ^{1}H NMR spectrum of **5a** ($CDCl_3$, 300 MHz).

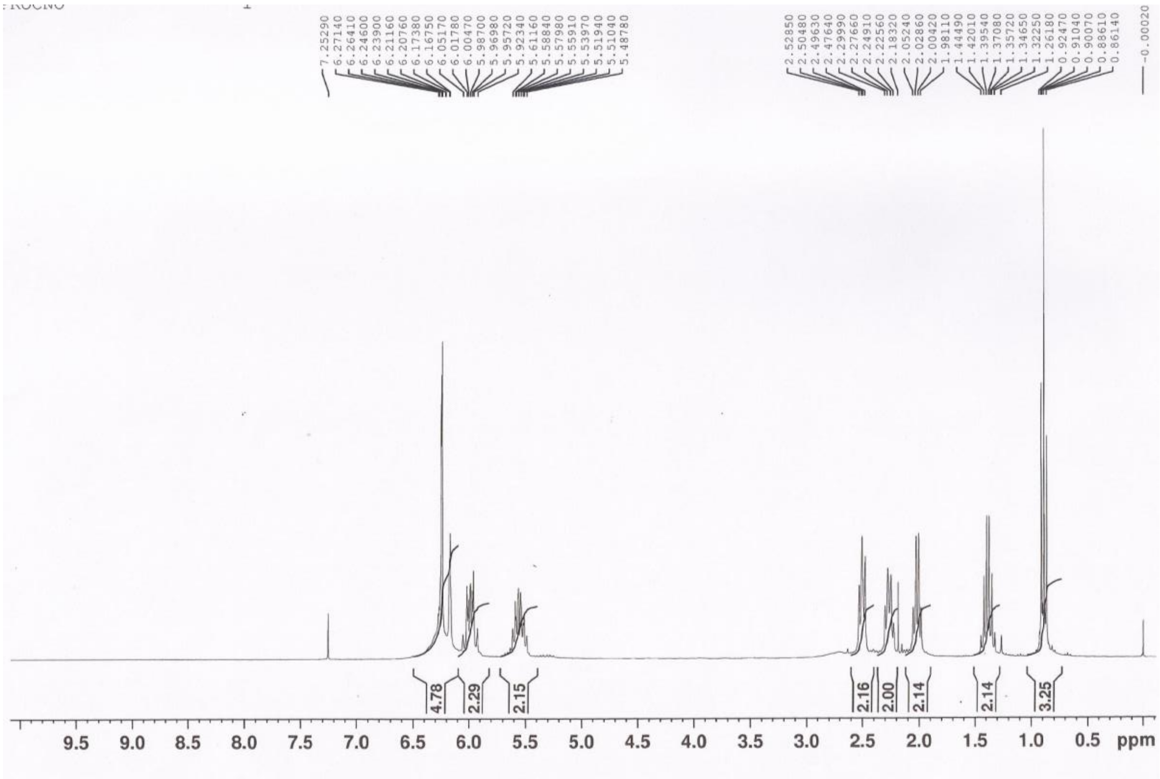

Figure S30. ^{13}C NMR spectrum of **5a** ($CDCl_3$, 75 MHz).

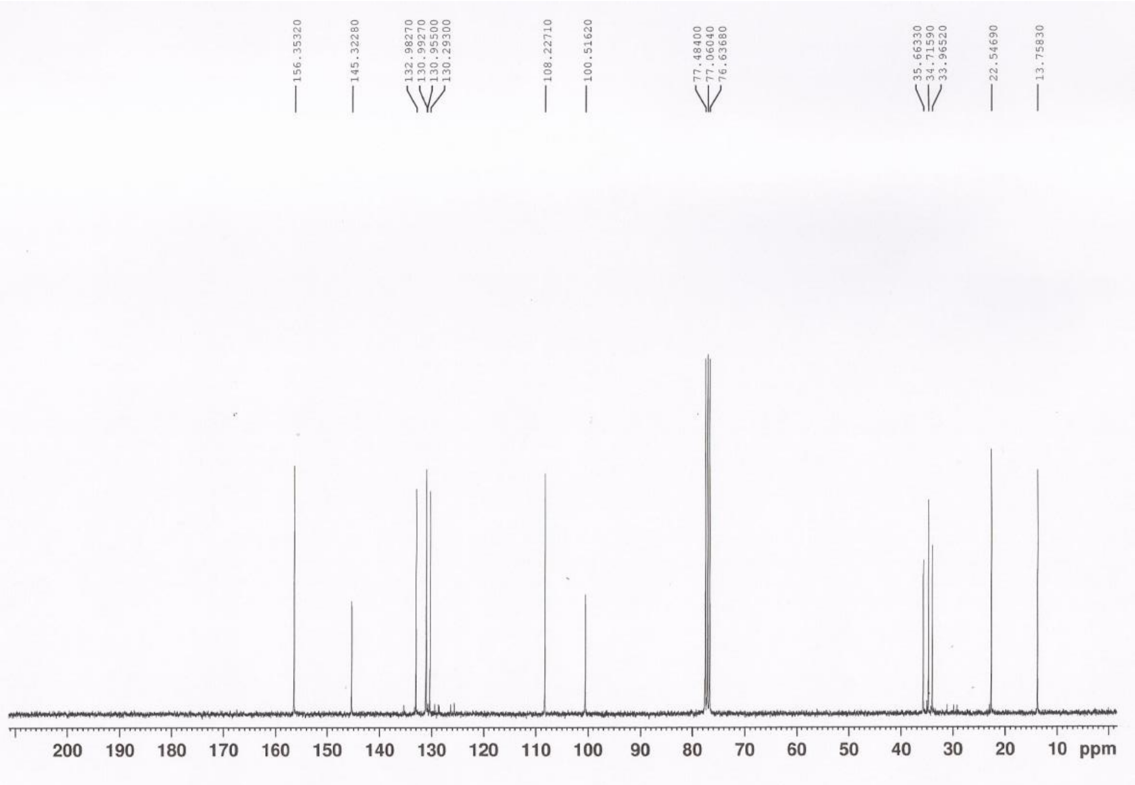

Figure S31. COSY spectrum of **5a** ($CDCl_3$, 300 MHz).

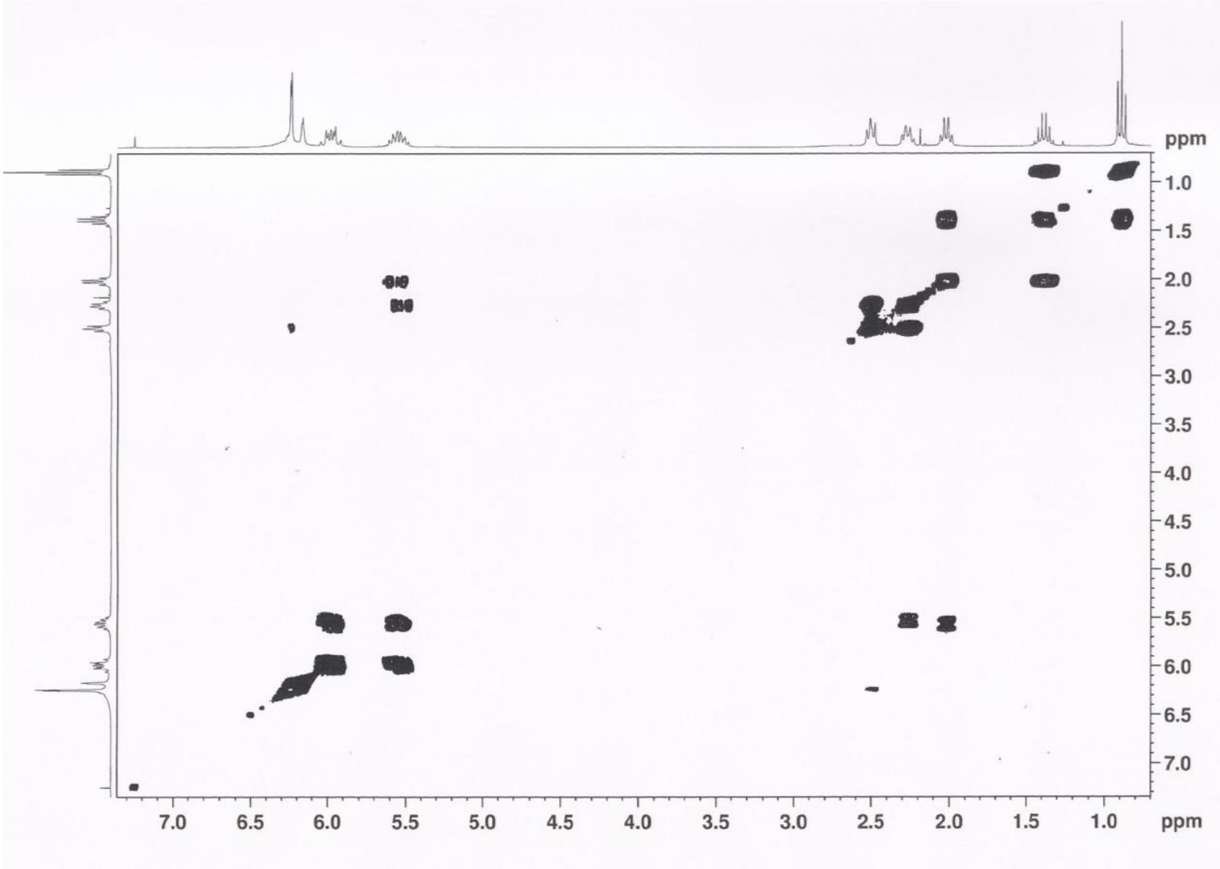

Figure S32. HSQC spectrum of **5a** ($CDCl_3$, 300 MHz).

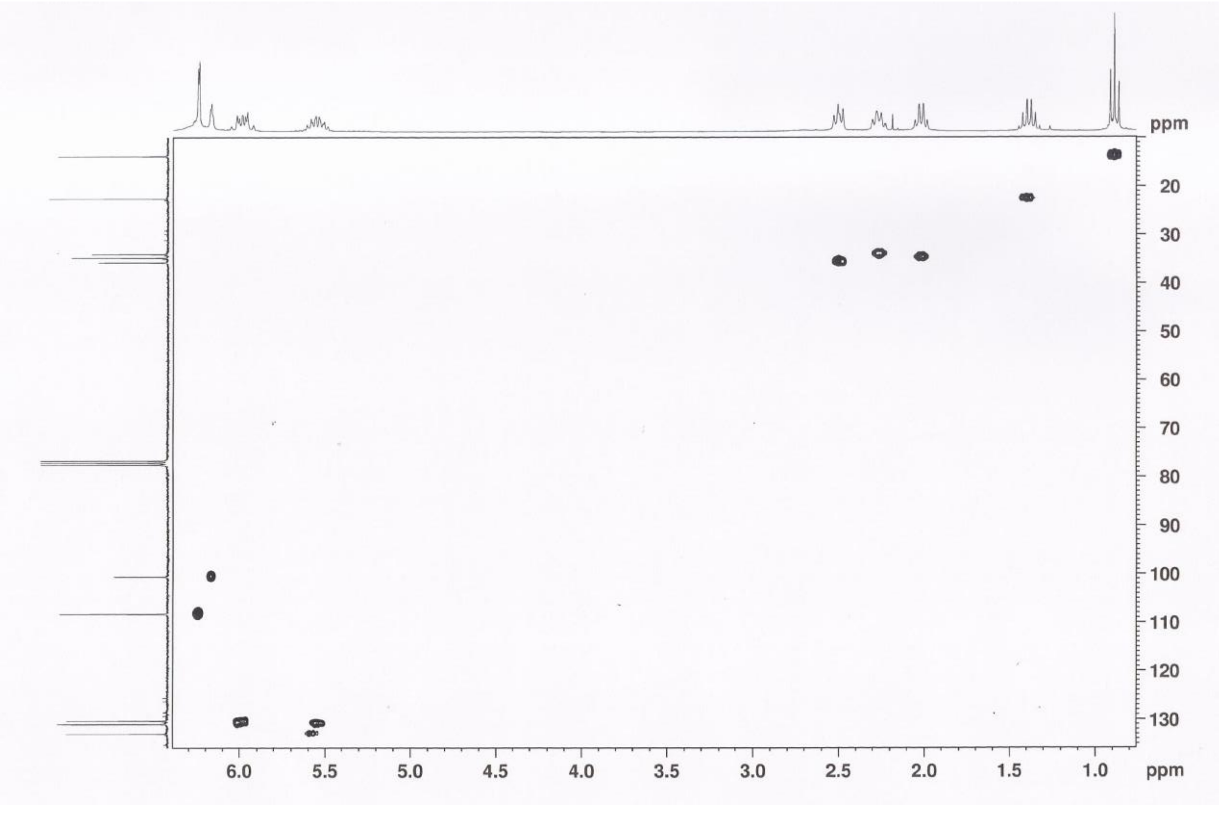

Figure S33. HMBC spectrum of **5a** ($CDCl_3$, 300 MHz).

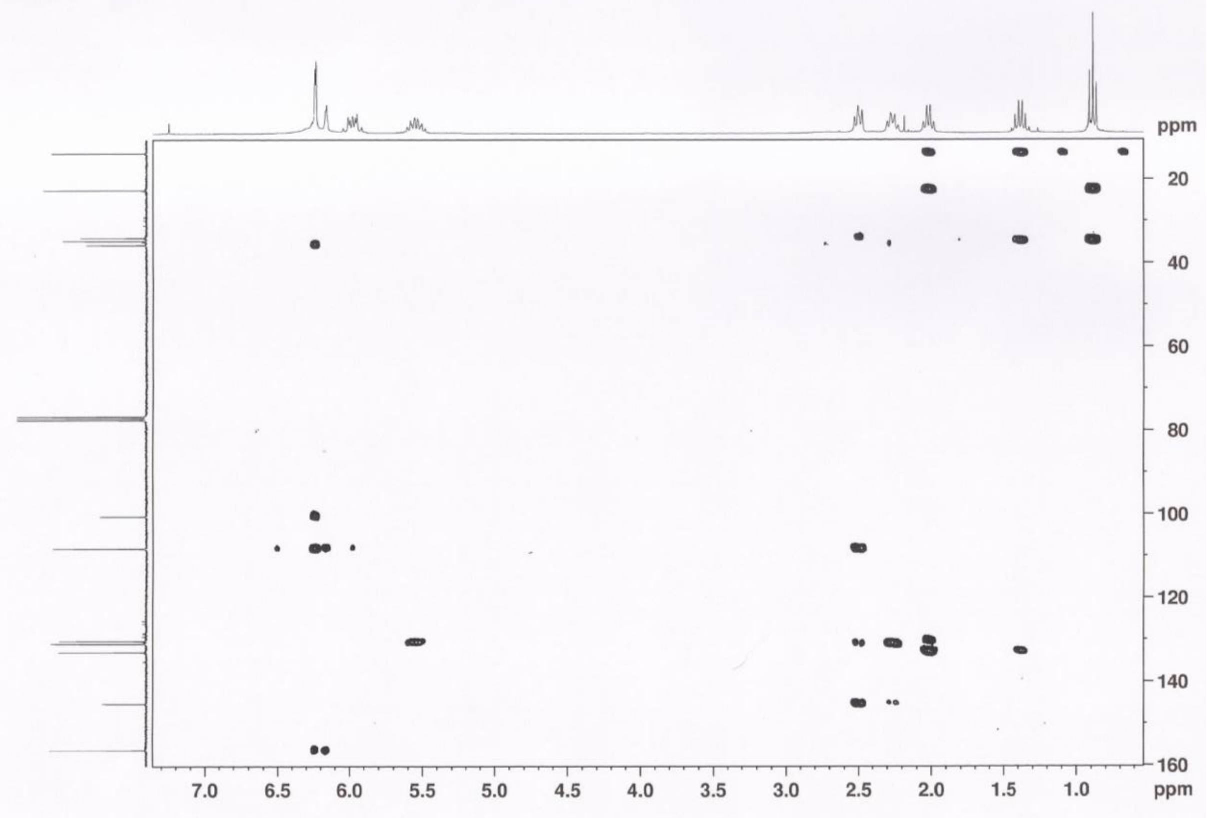

Figure S34. ^{1}H NMR spectrum of **5b** (DMSO-*d6*, 300 MHz).

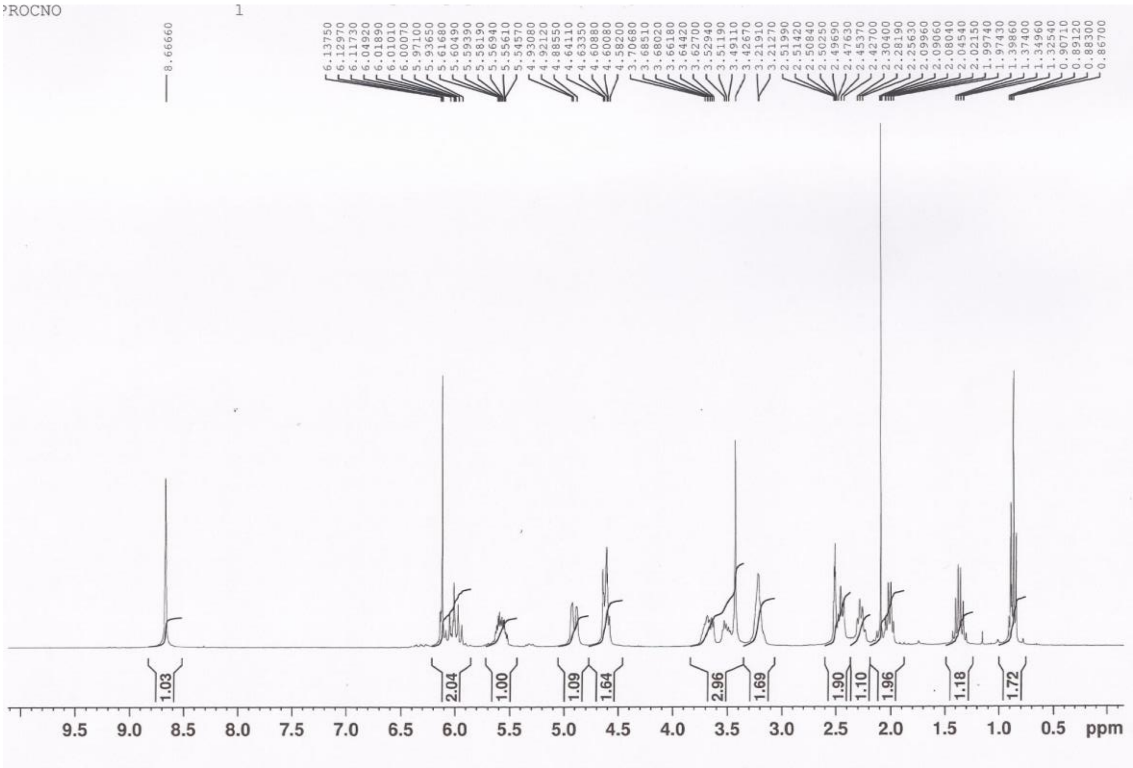

Figure S35. ^{13}C NMR spectrum of **5b** (DMSO-*d6*, 75 MHz).

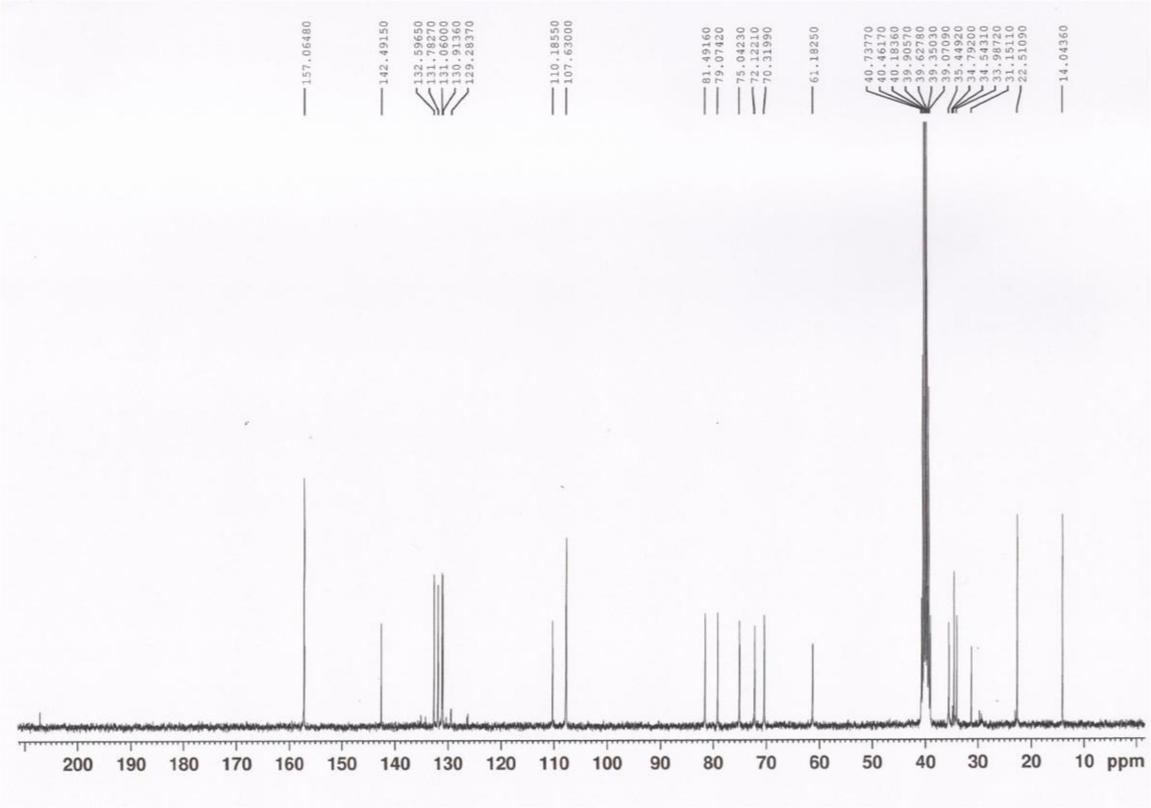

Figure S36. COSY spectrum of **5b** (DMSO-*d6*, 300 MHz).

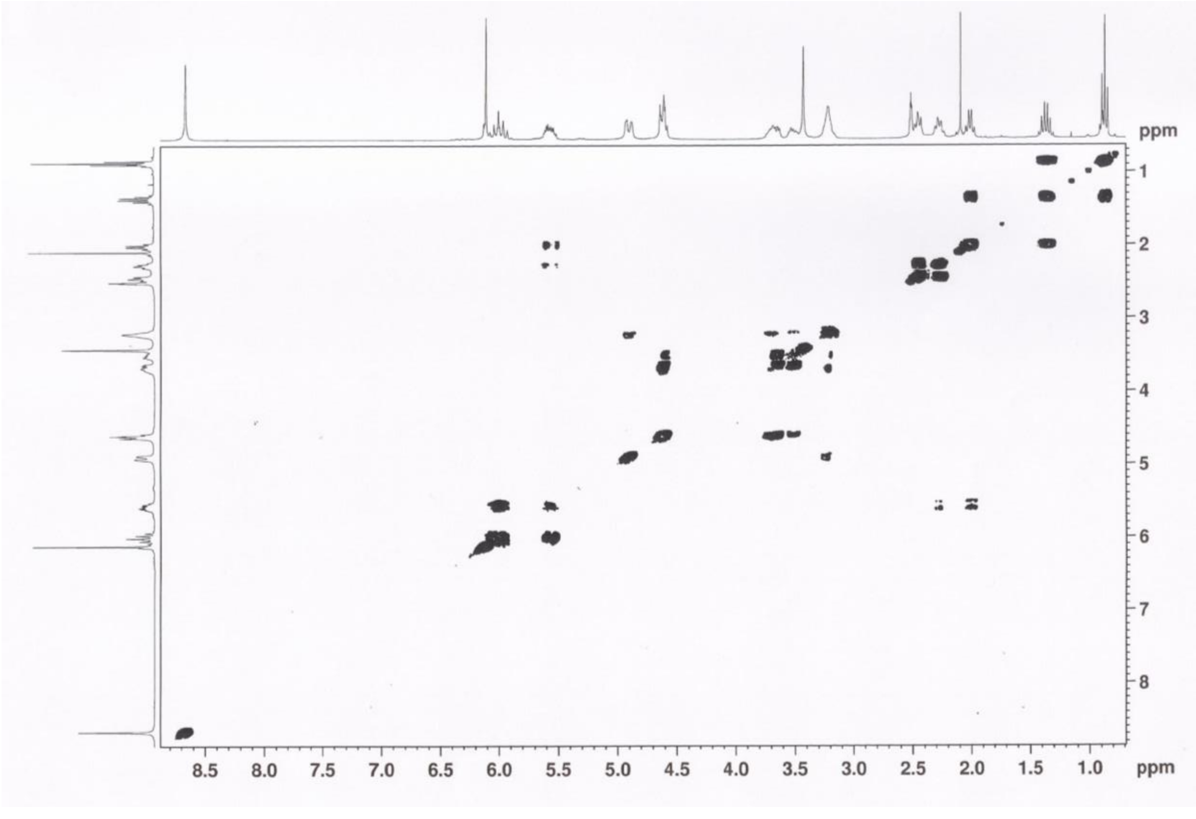

Figure S37. HSQC spectrum of **5b** (DMSO-*d6*, 300 MHz).

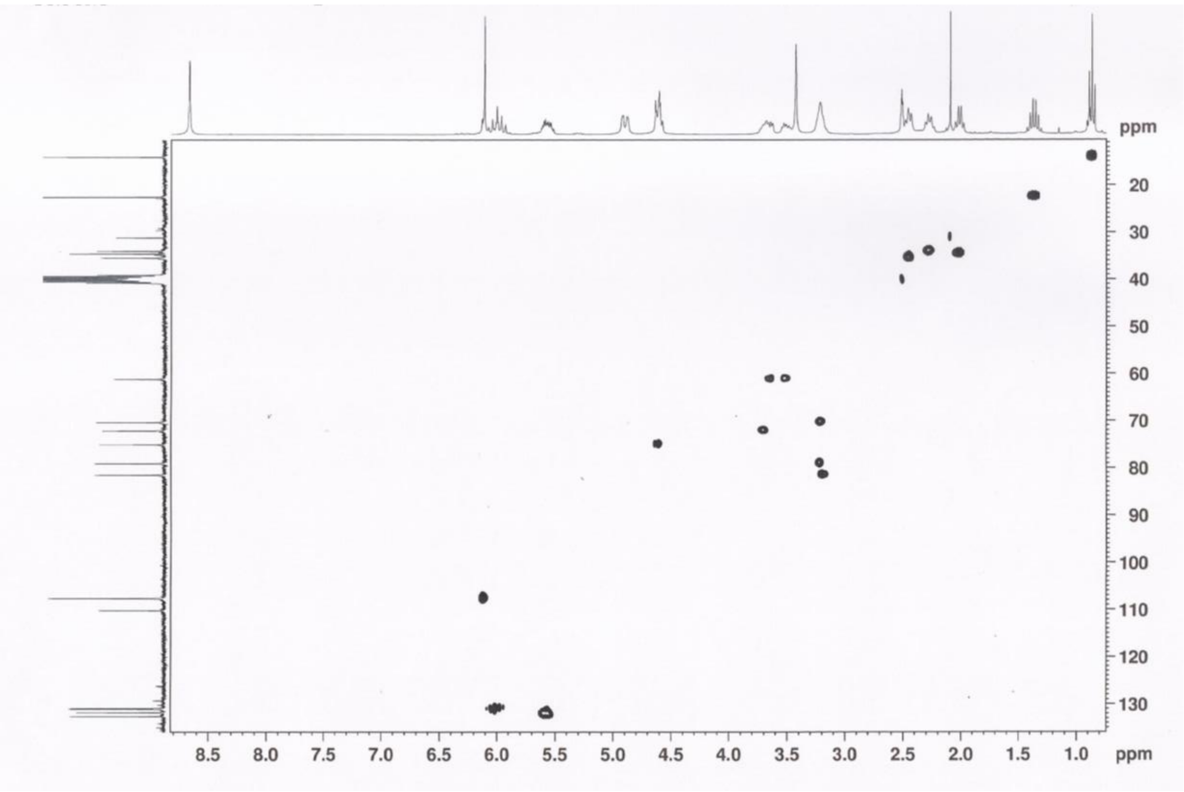

Figure S38. HMBC spectrum of **5b** (DMSO-*d6*, 300 MHz).

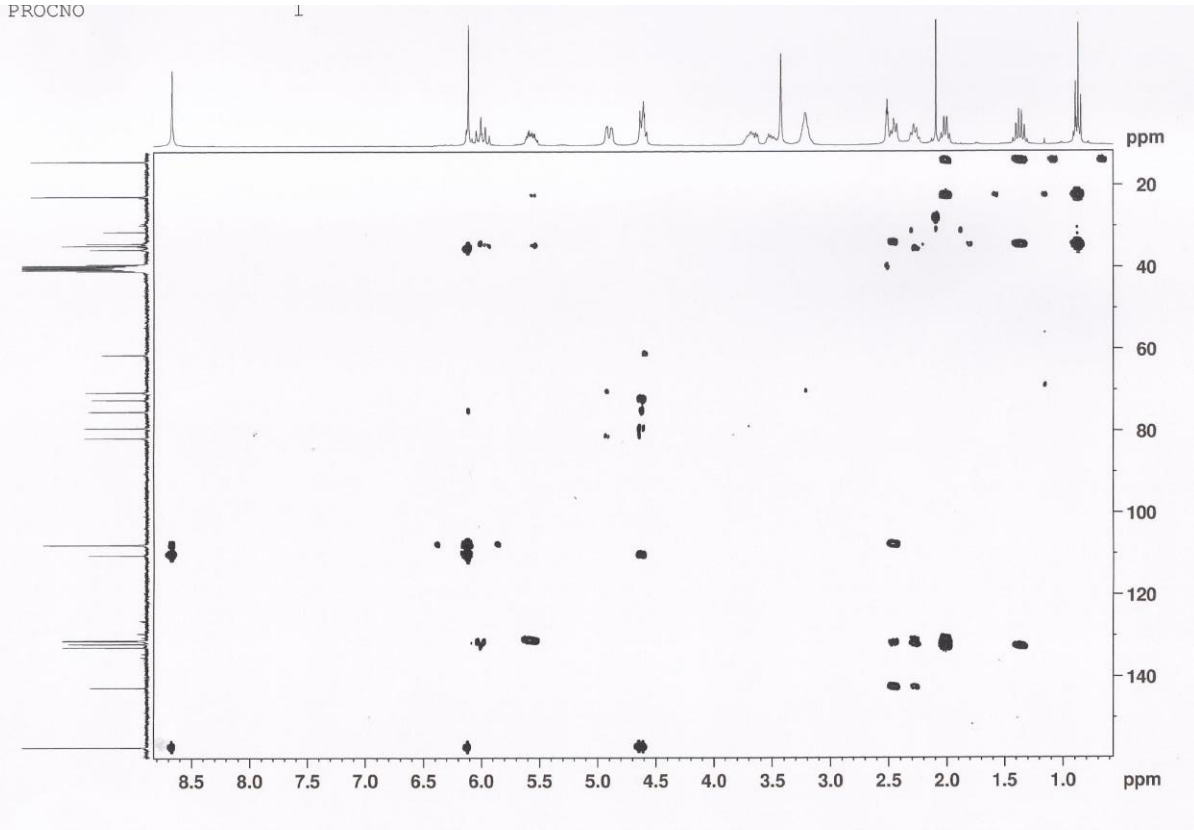

Figure S39. ^{1}H NMR spectrum of **5c** (DMSO-*d6*, 500 MHz).

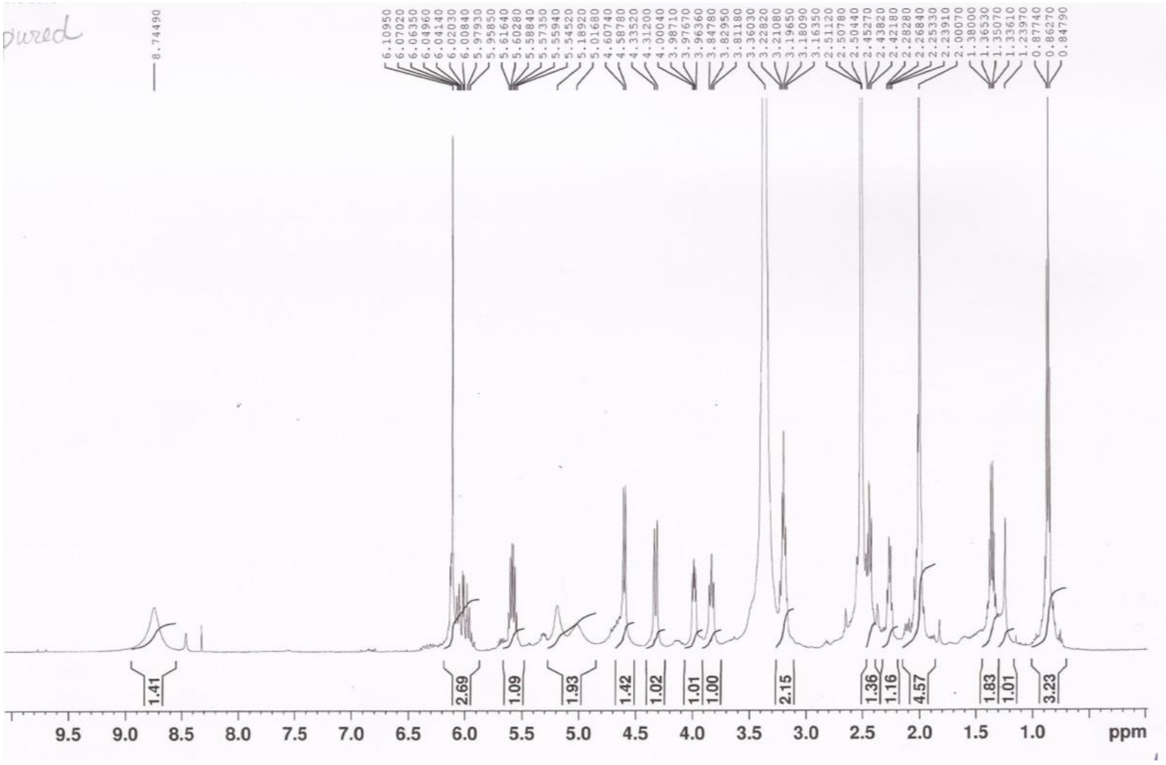

Figure S40. ^{13}C NMR spectrum of **5c** (DMSO-*d6*, 125 MHz)

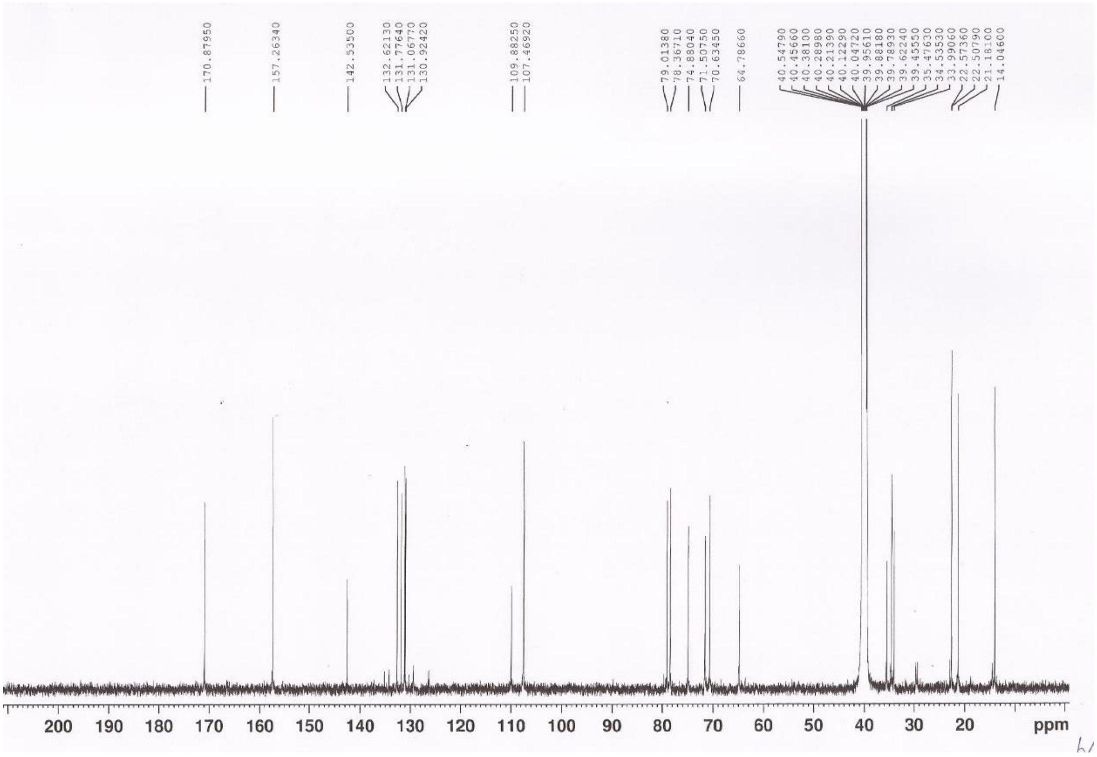

Figure S41. COSY spectrum of **5c** (DMSO-*d6*, 500 MHz)

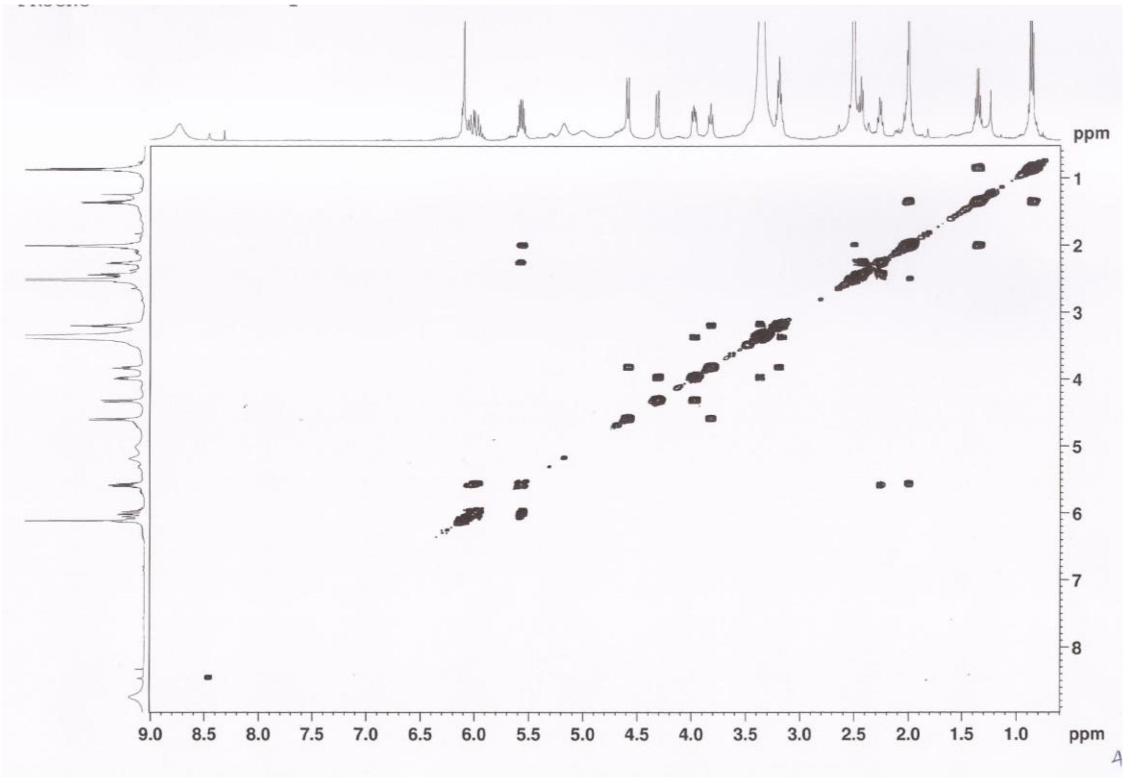

Figure S42. HSQC spectrum of **5c** (DMSO-*d6*, 500 MHz).

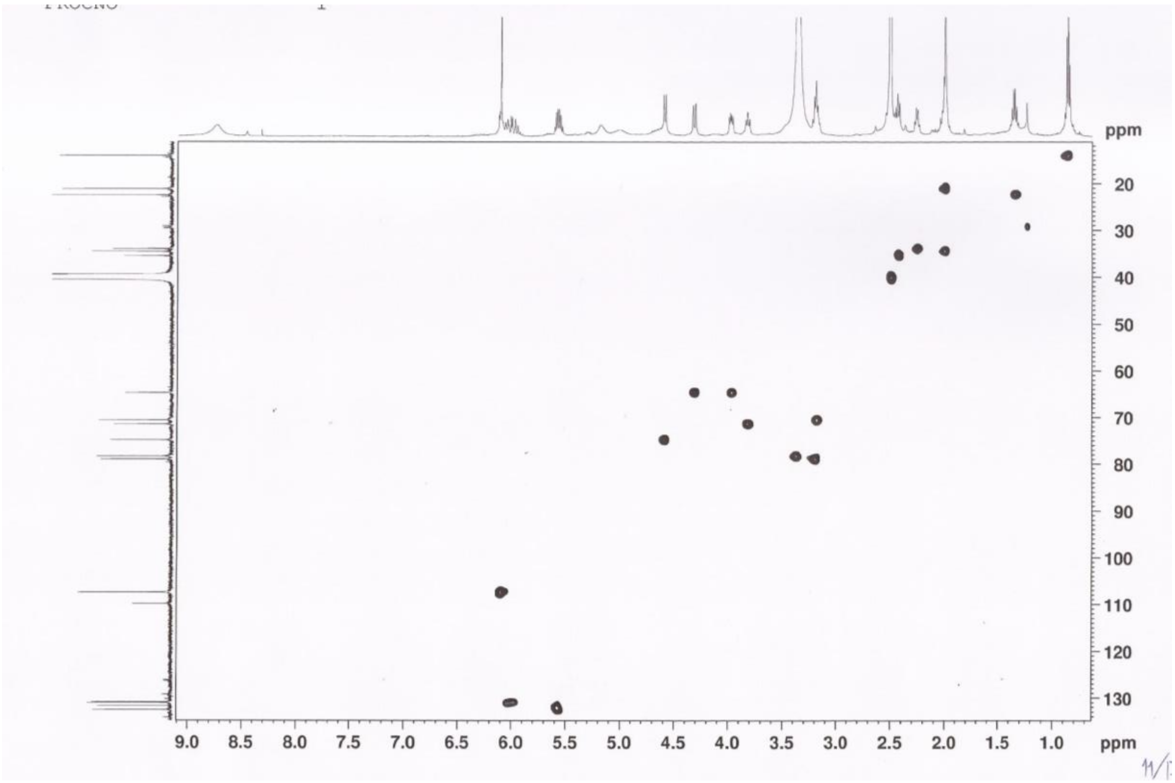

Figure S43. HMBC spectrum of **5c** (DMSO-d_6, 500 MHz).

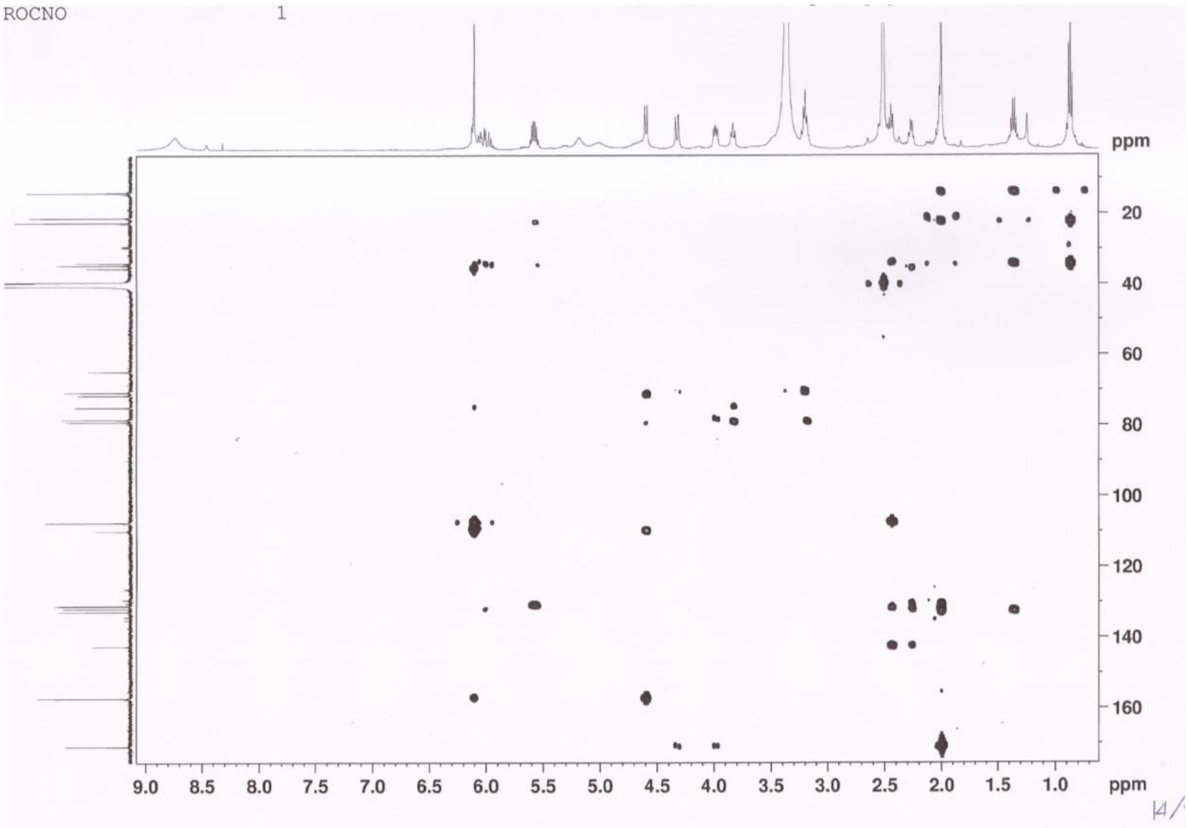

Figure S44. ^{1}H NMR spectrum of **5c** (DMSO-d_6, 300 MHz).

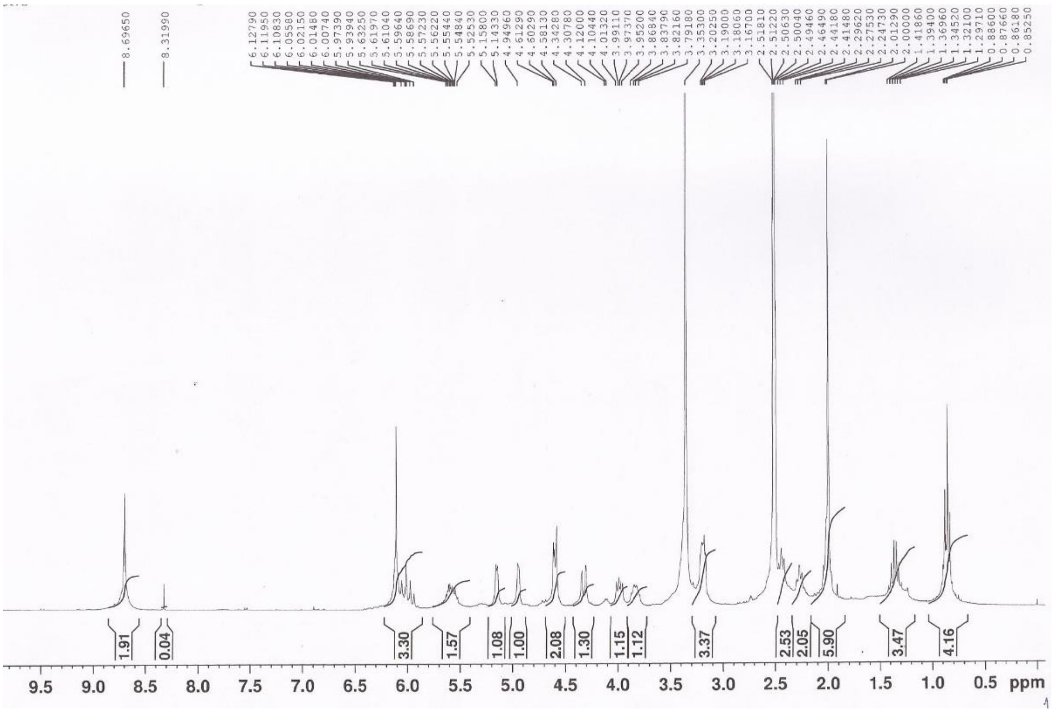

Figure S45. COSY spectrum of **5c** (DMSO-d_6, 300 MHz).

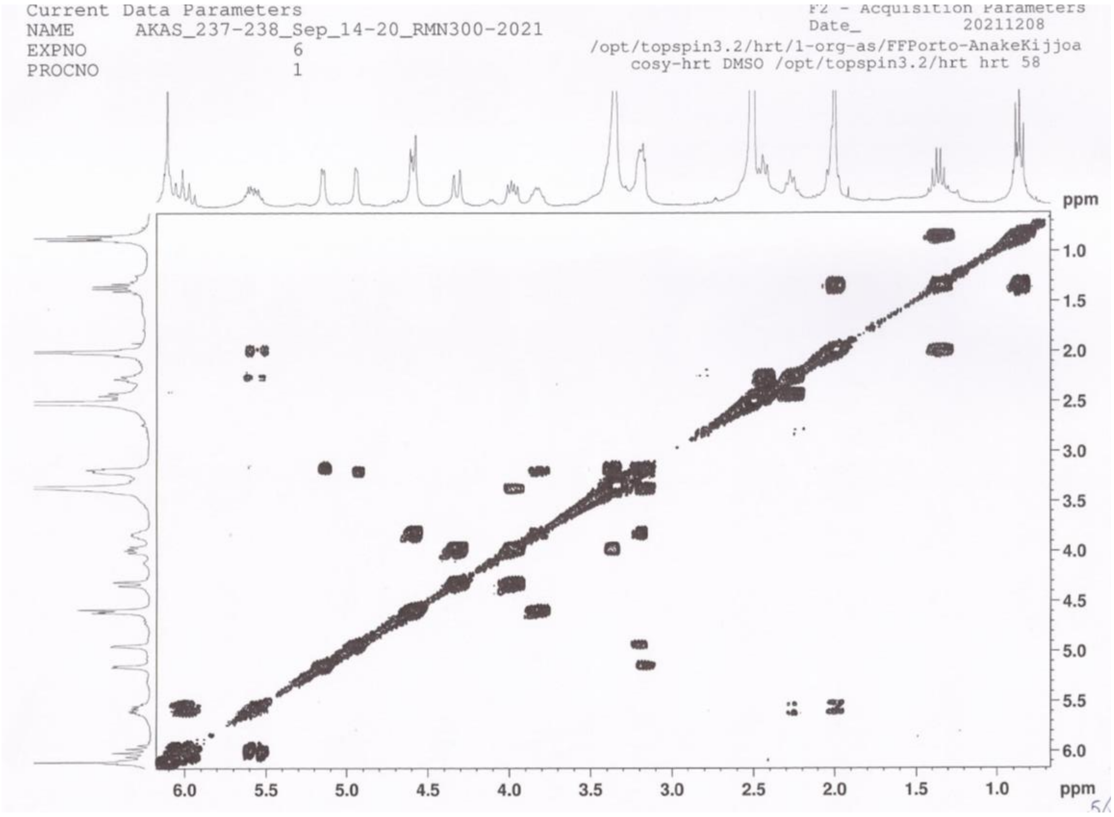

Figure S46. HMBC spectrum of **5c** (DMSO-d_6, 300 MHz).

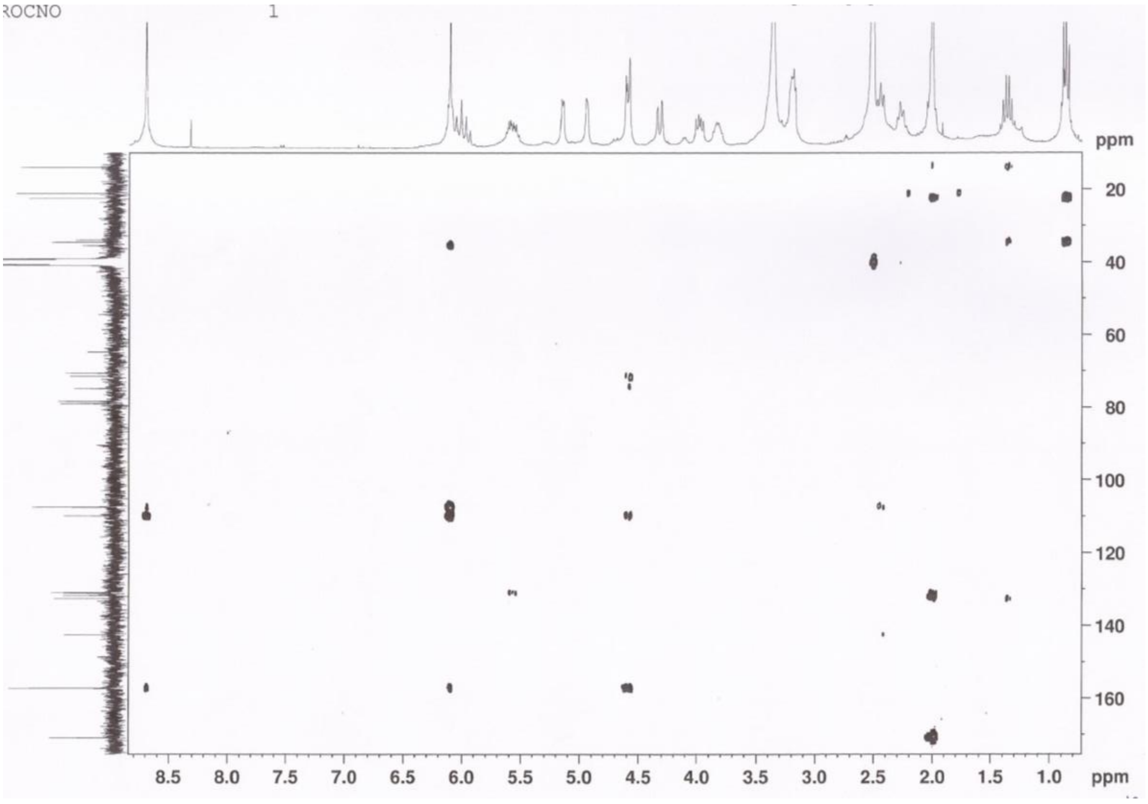

Figure S47. ^{1}H NMR spectrum of **6a** ($CDCl_3$, 300 MHz).

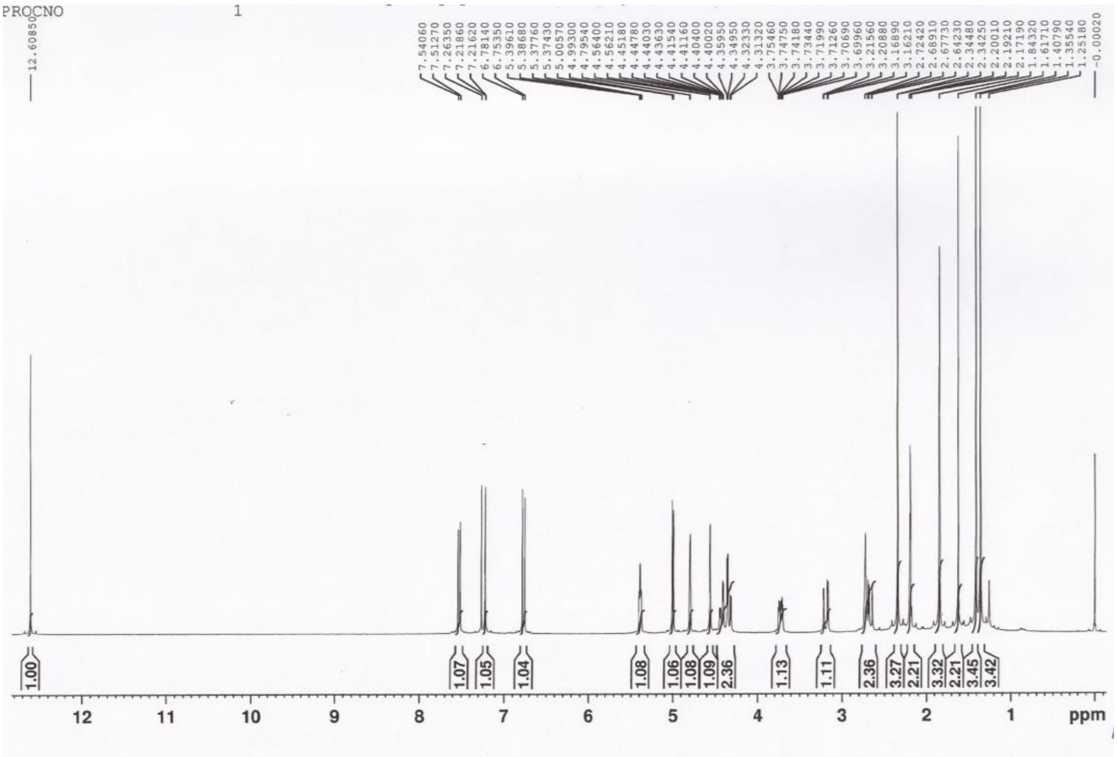

Figure S48. ^{13}C NMR spectrum of **6a** ($CDCl_3$, 75 MHz).

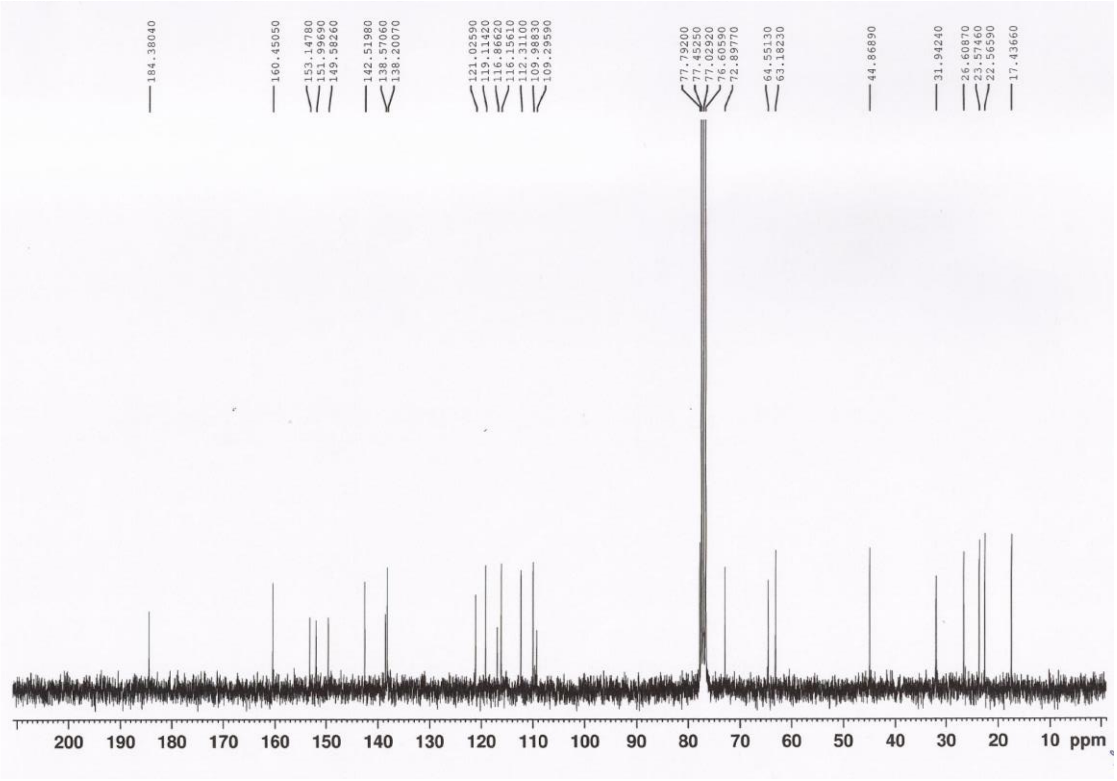

Figure S49. COSY spectrum of **6a** ($CDCl_3$, 300 MHz).

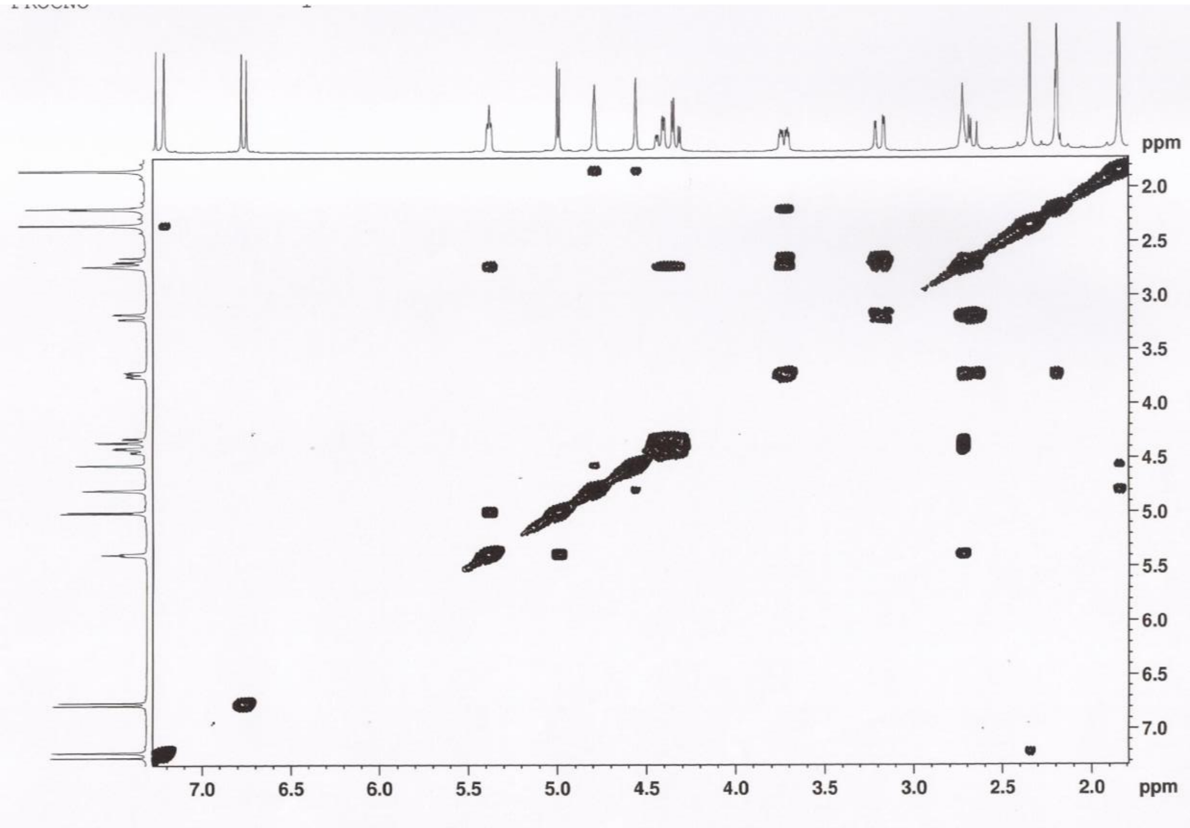

Figure S50. HSQC spectrum of **6a** ($CDCl_3$, 300 MHz).

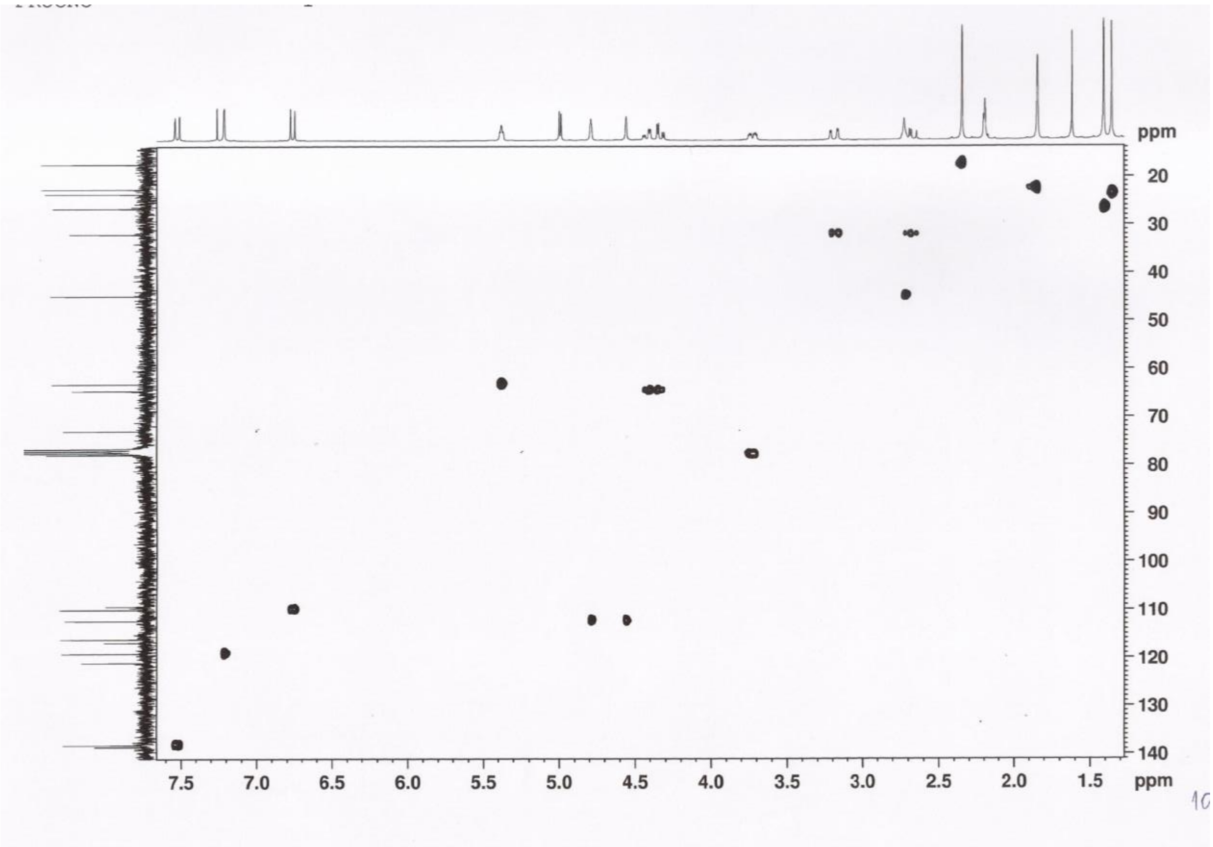

Figure S51. HMBC spectrum of **6a** ($CDCl_3$, 300 MHz).

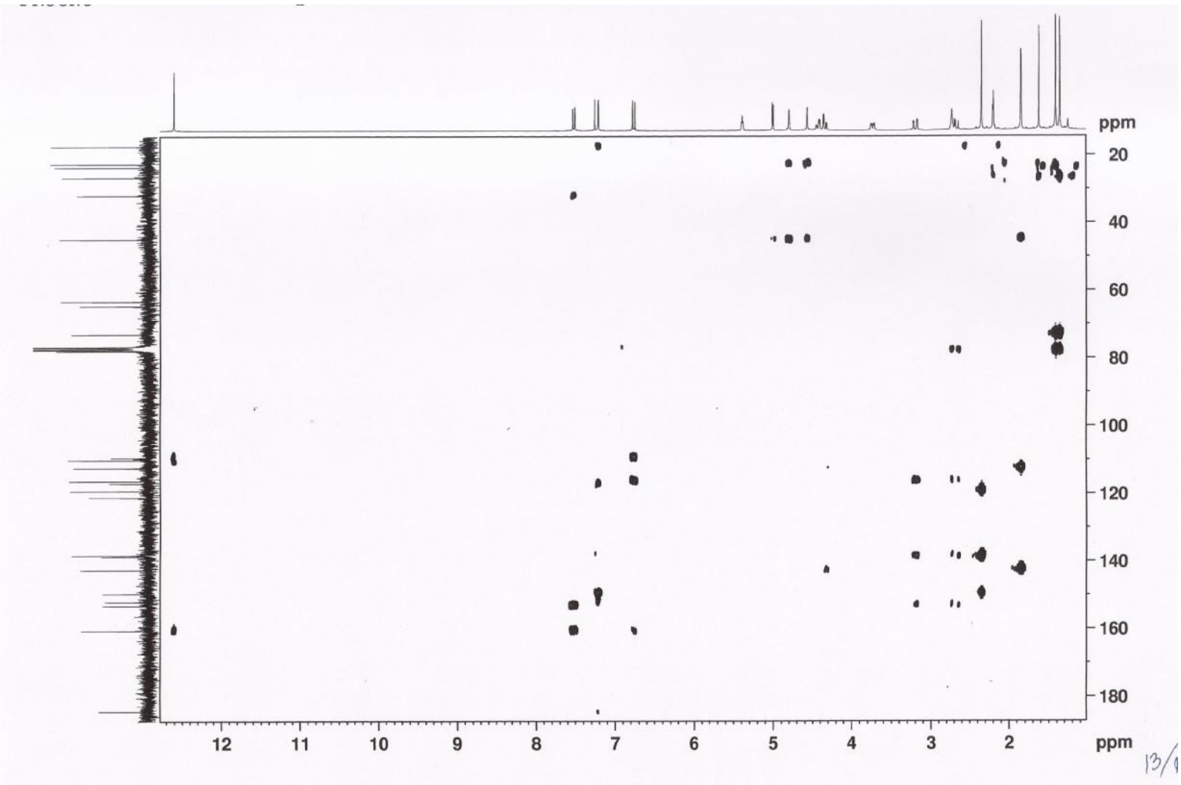

Figure S52. ^{1}H NMR spectrum of **6b** ($CDCl_3$, 300 MHz).

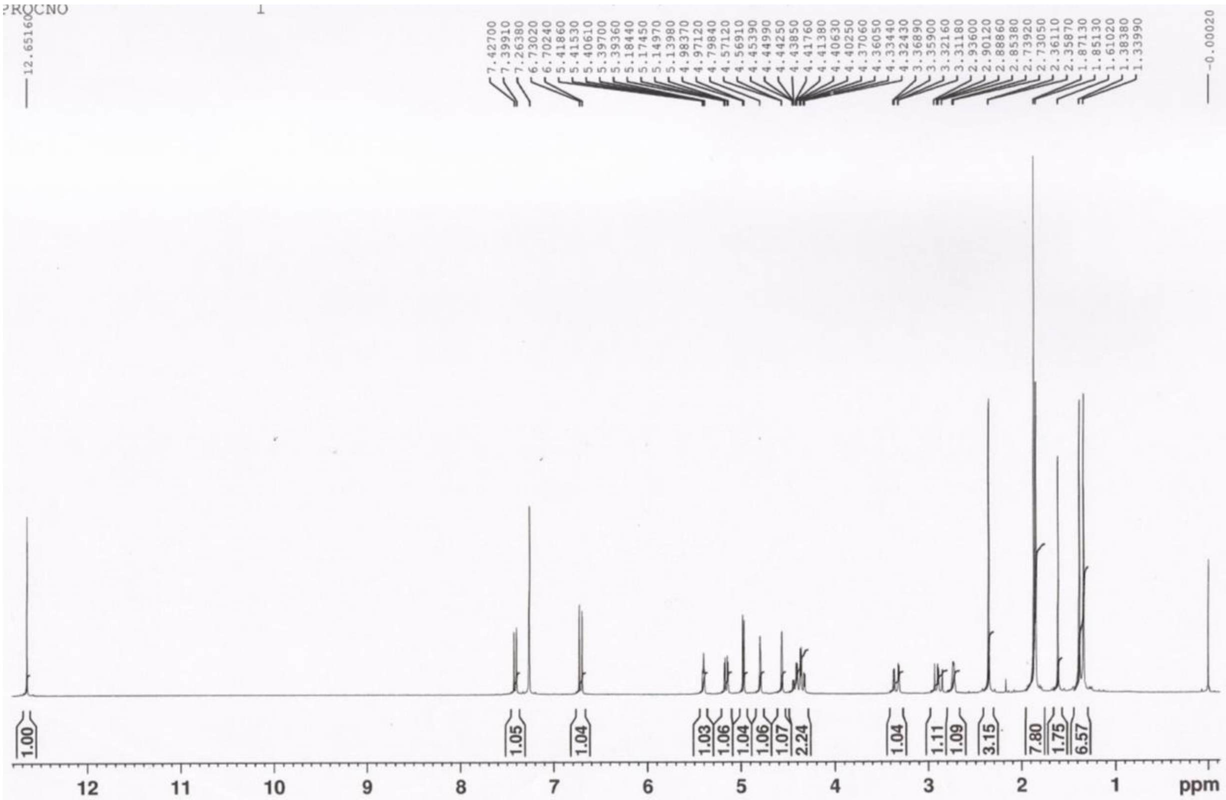

Figure S53. ^{13}C NMR spectrum of **6b** ($CDCl_3$, 75 MHz).

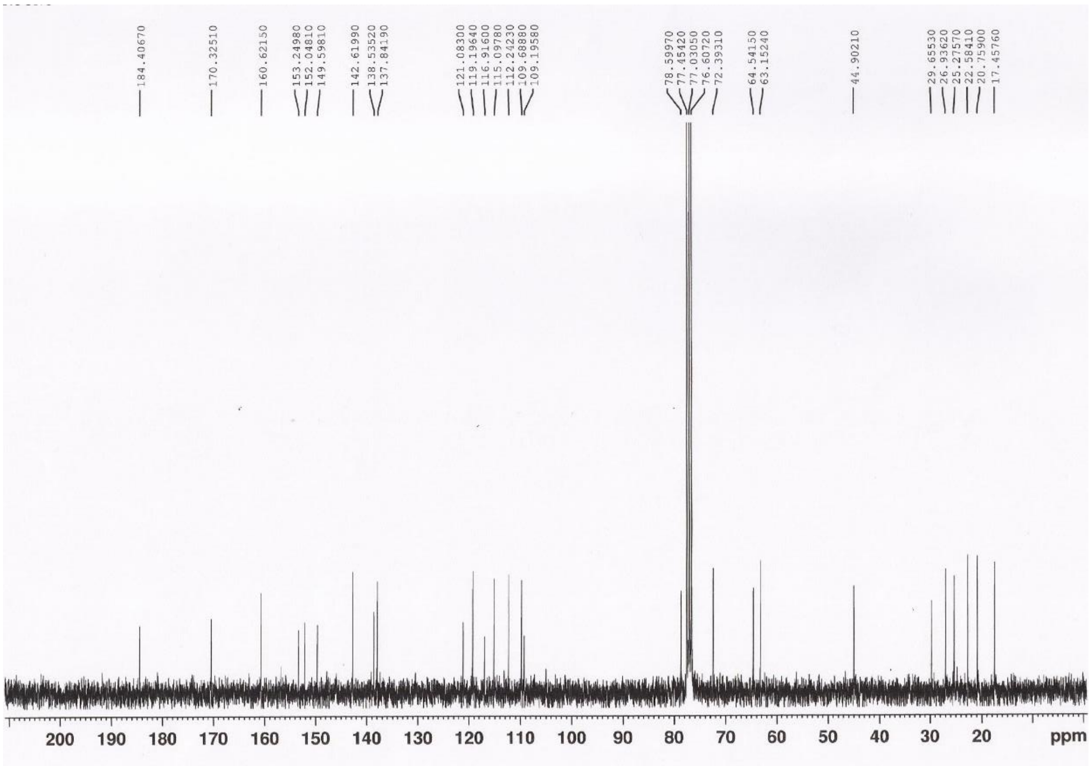

Figure S54. COSY spectrum of **6b** ($CDCl_3$, 300 MHz).

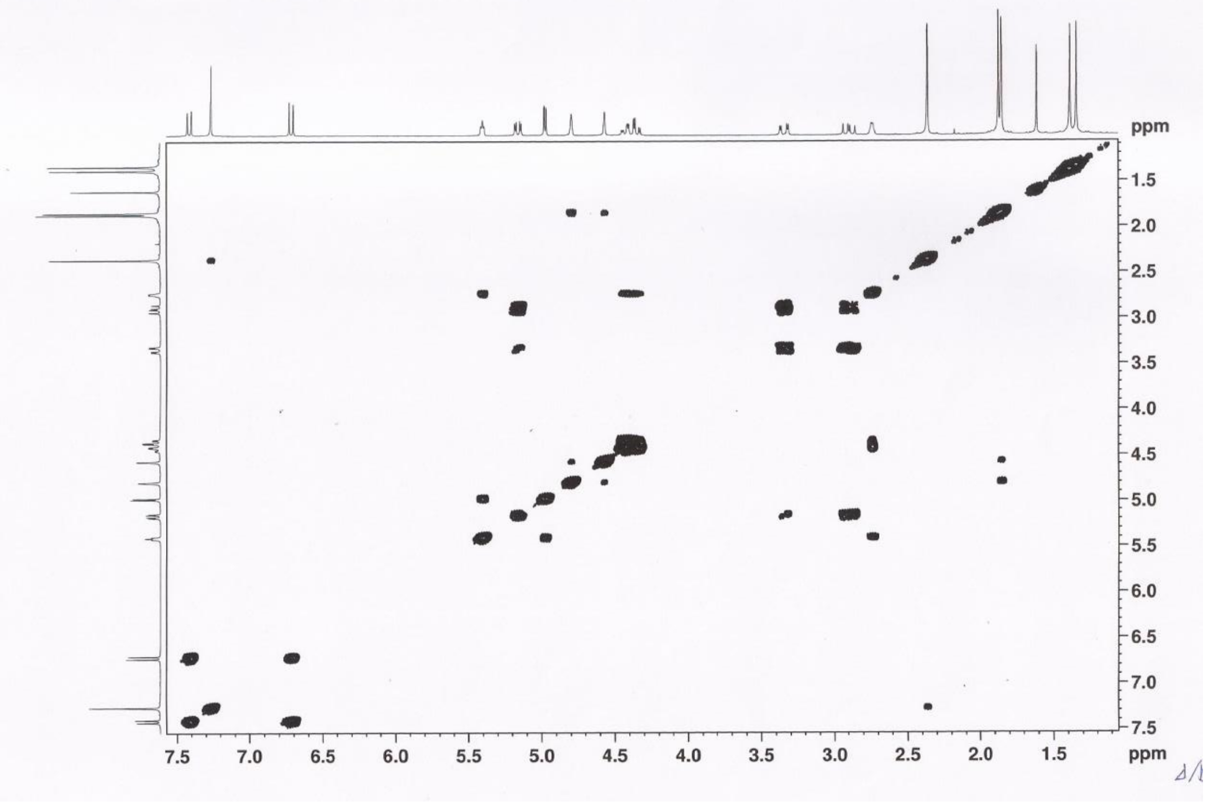

Figure S55. HSQC spectrum of **6b** ($CDCl_3$, 300 MHz).

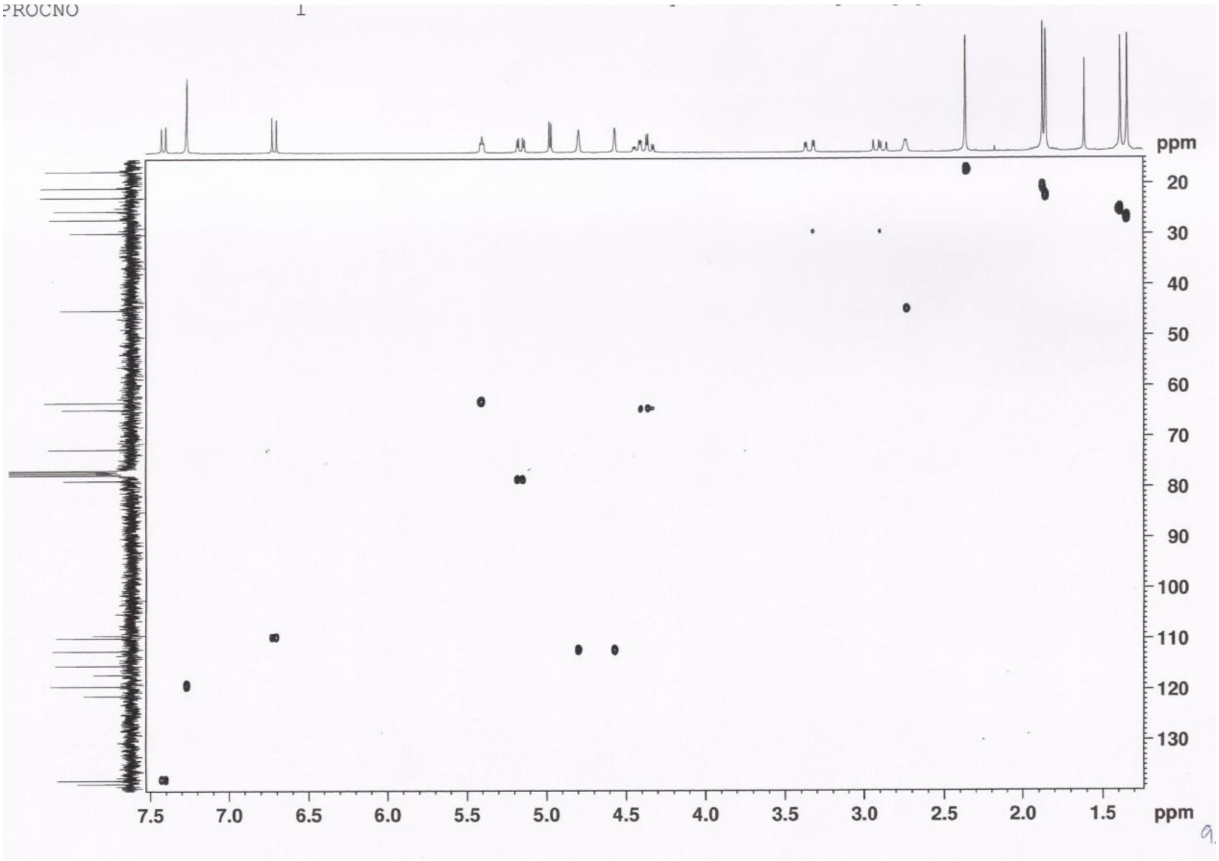

Figure S56. HMBC spectrum of **6b** ($CDCl_3$, 300 MHz).

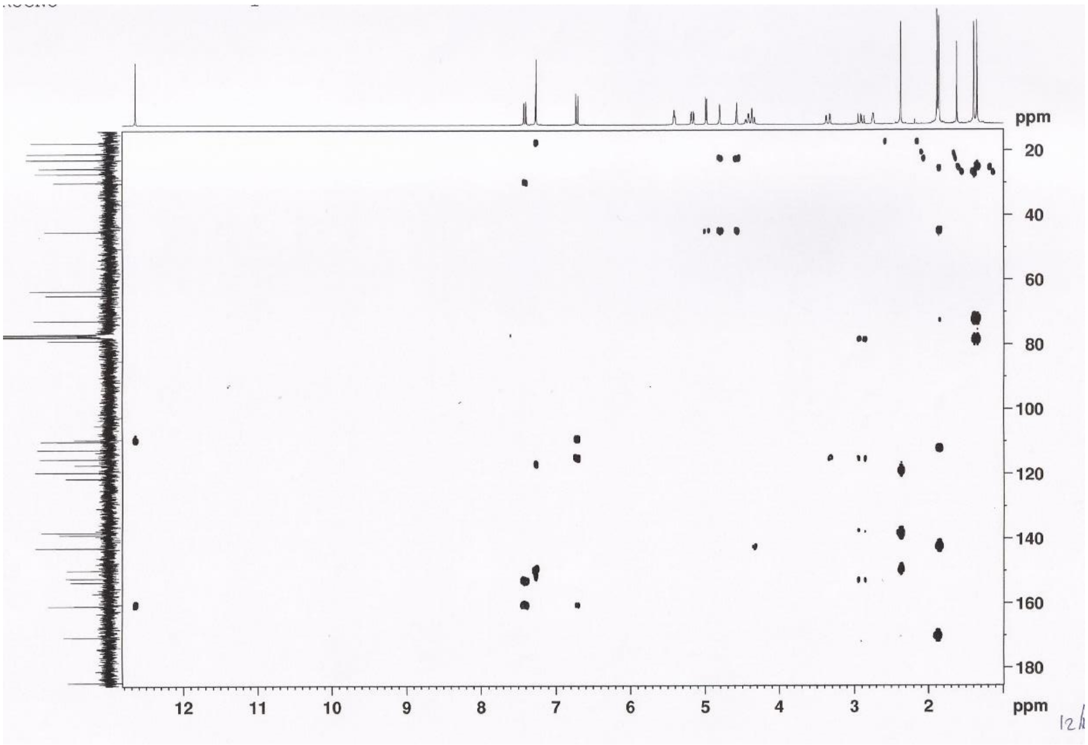

Figure S57. (+)-HRESIMS of **2**.

Elemental Composition Report [MH]$^+$

Single Mass Analysis
Tolerance = 5.0 PPM / DBE: min = -1.5, max = 100.0
Element prediction: Off
Number of isotope peaks used for i-FIT = 3

Monoisotopic Mass, Even Electron Ions
14 formula(e) evaluated with 1 results within limits (up to 100 closest results for each mass)
Elements Used:
C: 11-11 H: 0-150 O: 0-50

Minimum:				-1.5				
Maximum:		5.0	5.0	100.0				
Mass	Calc. Mass	mDa	PPM	DBE	i-FIT	Norm	Conf(%)	Formula
225.0765	225.0763	0.2	0.9	5.5	522.0	n/a	n/a	C11 H13 O5

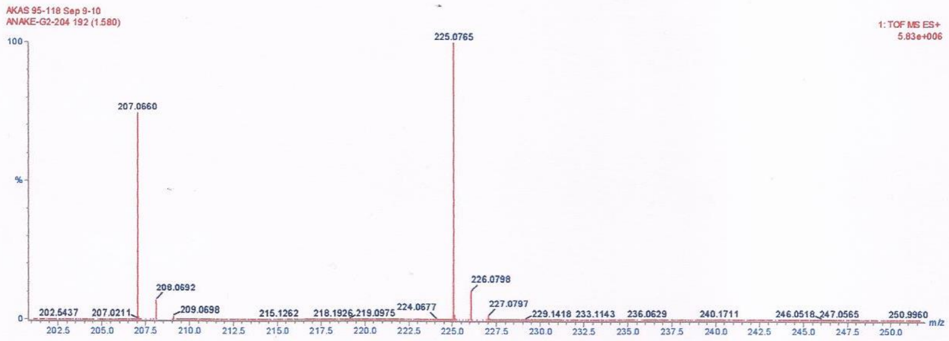

Figure S58. (+)-HRESIMS of **4**.

Match for $C_{23}H_{19}NO_5$

Elemental Composition Report [MH]$^+$

Single Mass Analysis
Tolerance = 5.0 PPM / DBE: min = -1.5, max = 100.0
Element prediction: Off
Number of isotope peaks used for i-FIT = 3

Monoisotopic Mass, Even Electron Ions
364 formula(e) evaluated with 1 results within limits (up to 100 closest results for each mass)
Elements Used:
C: 23-23 H: 0-150 N: 0-50 O: 0-50

Minimum:				-1.5				
Maximum:		5.0	5.0	100.0				
Mass	Calc. Mass	mDa	PPM	DBE	i-FIT	Norm	Conf(%)	Formula
390.1340	390.1341	-0.1	-0.3	14.5	706.9	n/a	n/a	C23 H20 N O5

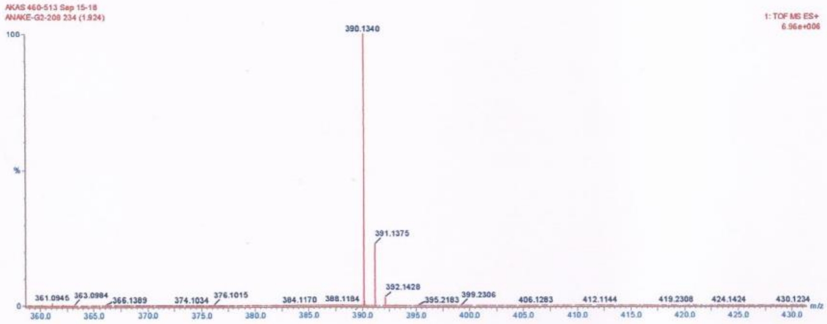

Figure S59. (+)-HRESIMS of **5a**.

Elemental Composition Report [MH]+

Single Mass Analysis
Tolerance = 5.0 PPM / DBE: min = -1.5, max = 100.0
Element prediction: Off
Number of isotope peaks used for i-FIT = 3

Monoisotopic Mass, Even Electron Ions
15 formula(e) evaluated with 1 results within limits (up to 100 closest results for each mass)
Elements Used:
C: 15-15 H: 0-150 O: 0-50

Minimum:				-1.5				
Maximum:		5.0	5.0	100.0				
Mass	Calc. Mass	mDa	PPM	DBE	i-FIT	Norm	Conf(%)	Formula
233.1542	233.1542	0.0	0.0	5.5	932.2	n/a	n/a	C15 H21 O2

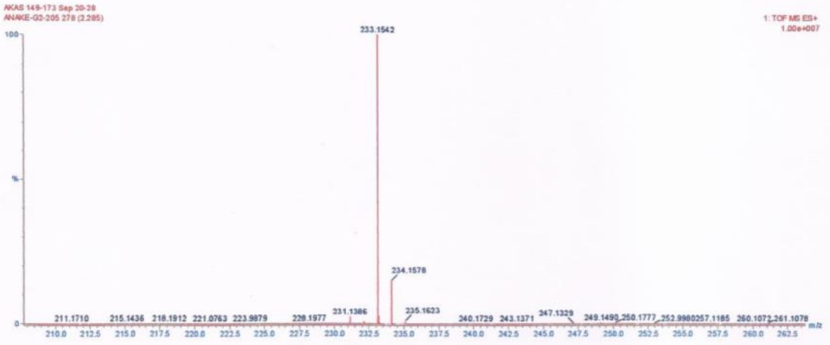

Figure S60. (+)-HRESIMS of **5b**.

Match for $C_{21}H_{30}O_7$

Elemental Composition Report [MH]+

Single Mass Analysis
Tolerance = 5.0 PPM / DBE: min = -1.5, max = 100.0
Element prediction: Off
Number of isotope peaks used for i-FIT = 3

Monoisotopic Mass, Even Electron Ions
25 formula(e) evaluated with 1 results within limits (up to 100 closest results for each mass)
Elements Used:
C: 21-21 H: 0-150 O: 0-50

Minimum:				-1.5				
Maximum:		5.0	5.0	100.0				
Mass	Calc. Mass	mDa	PPM	DBE	i-FIT	Norm	Conf(%)	Formula
395.2076	395.2070	0.6	1.5	6.5	816.8	n/a	n/a	C21 H31 O7

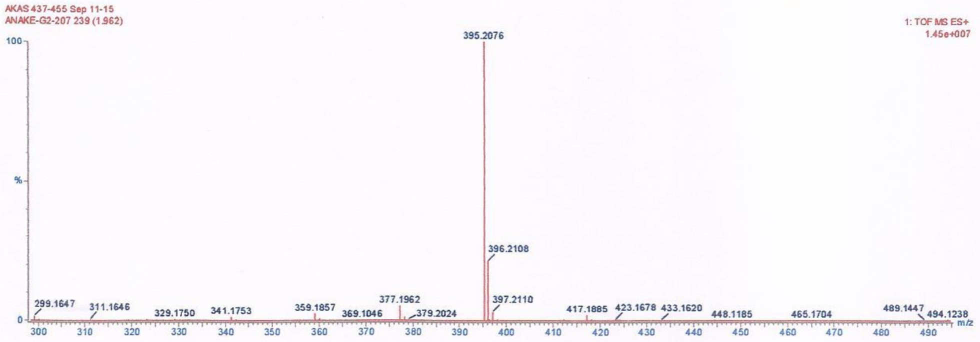

Figure S61. (+)-HRESIMS of **5c**.

Elemental Composition Report [MH]+

Single Mass Analysis
Tolerance = 5.0 PPM / DBE: min = -1.5, max = 100.0
Element prediction: Off
Number of isotope peaks used for i-FIT = 3
Monoisotopic Mass, Odd and Even Electron Ions
28 formula(e) evaluated with 1 results within limits (up to 100 closest results for each mass)
Elements Used:
C: 23-23 H: 0-150 O: 0-50

Minimum:				-1.5				
Maximum:		5.0	5.0	100.0				
Mass	Calc. Mass	mDa	PPM	DBE	i-FIT	Norm	Conf(%)	Formula
437.2175	437.2175	0.0	0.0	7.5	414.2	n/a	n/a	C23 H33 O8

Elemental Composition Report [MNa]+

Single Mass Analysis
Tolerance = 5.0 PPM / DBE: min = -1.5, max = 100.0
Element prediction: Off
Number of isotope peaks used for i-FIT = 3
Monoisotopic Mass, Odd and Even Electron Ions
57 formula(e) evaluated with 1 results within limits (up to 100 closest results for each mass)
Elements Used:
C: 23-23 H: 0-150 O: 0-50 Na: 0-1

Minimum:				-1.5				
Maximum:		5.0	5.0	100.0				
Mass	Calc. Mass	mDa	PPM	DBE	i-FIT	Norm	Conf(%)	Formula
459.1989	459.1995	-0.6	-1.3	7.5	629.7	n/a	n/a	C23 H32 O8 Na

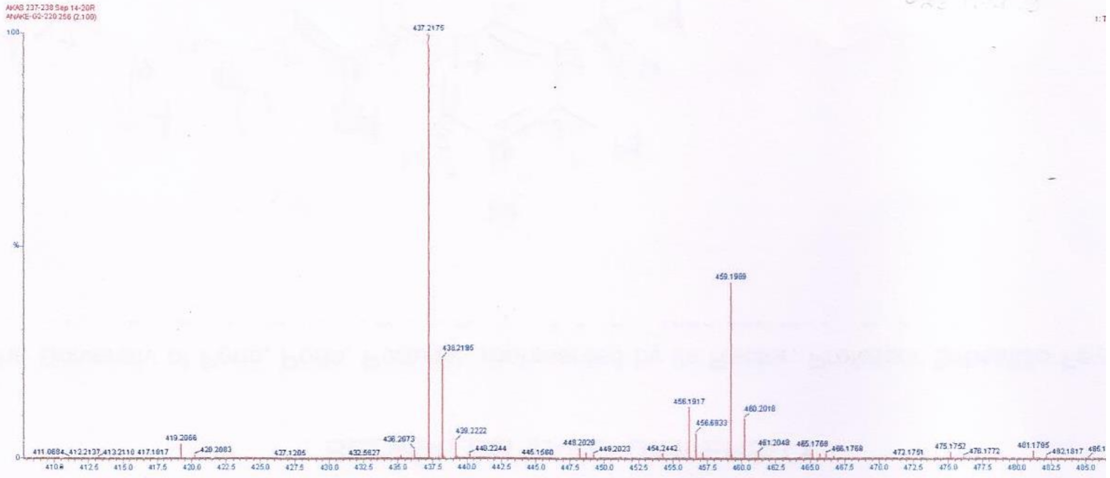

Table S1. ^{1}H and ^{13}C NMR data of **3a** and **3b** ($CDCl_3$, 300 and 75 MHz).

**3a**			**3b**	
Position	δ_C, type	δ_H, (*J* in Hz)	δ_C, type	δ_H, (*J* in Hz)
1	165.3, C		151.9, C	
2	104.7, CH	6.78, d (2.3(	144.9, C	
3	163.0, C		147.7, C	
4	103.9, CH	7.46, d (2.5)	106.6, CH	7.70, s
4a	132.3, C		127.8, C	
5	120.0, CH	7.57, dd (1.7, 0.5)	120.2, CH	7.59, dd (1.6, 0.5)
6	146.9, C		147.7, C	
7	124.8, CH	7.08, dd (1.6, 0.8)	124.3, CH	7.08, dd (1.6, 0.8)
8	162.6, C		162.6, C	
8a	114.8, C		114.6, C	
9	187.5, CO		187.6, CO	
9a	115.2, C		120.2, C	
10	183.0, CO		182.0, C	
10a	137.7, C		132.5, C	
Me-11	22.0, CH_3	2.43,s	22.1, CH_3	2.44, s
OMe-1	56.1, CH_3	3.99, s	61.9, CH_3	4.09, s
OMe-3	56.3, CH_3	4.03, s	56.7, CH_3	4.03, s
OH-8	-	13.09, s		

Table S2. ^{1}H and ^{13}C NMR data of **5b** (DMSO-d_6, 300 and 75 MHz), **5c** (DMSO-d_6, 500 and 125 MHz) and **5a** ($CDCl_3$, 300 and 75 MHz).

	**5b**		**5c**		**5a**	
Position	δ_C, type	δ_H, (*J* in Hz)	δ_C, type	δ_H, (*J* in Hz)	δ_C, type	δ_H, (*J* in Hz)
1	157.1,C		157.3, HC		156.4, C	
2	110.2,C		109.9,C		100.5, CH	6.17, t (1.9)
3	157.1, C		157.3, C		156.4, C	
4	107.6, CH	6.11,s	107.5, CH	6.11, s	108.2, CH	6.24, d (2.1)
5	142.5, C		142.5, C		145.3, C	
6	107.6,CH	6.11,s	107.5, CH	6.11, s	108.2, CH	6.24, d (2.1)
7	35.5,CH_2	2.45, t (6.8)	35.5, CH_2	2.44 t (7.2)	35.7, CH_2	2.50, dd (9.1, 7.1)
8	34.0,CH_2	2.27, dd (14.3, 6.6)	34.0, CH_2	2.26, dd (14.7, 7.2)	34.7, CH_2	2.02,dd (14.4, 7.2)
9	131.8, CH	5.58, m	131.8, CH	5.59 ddd (14.6, 7.2, 7.2)	131.0, CH	5.55, m
10	130.9, CH	5.97,m	130.9, CH	5.97, m	130.3, CH	5.96, m
11	131.1, CH	6.04, m	131.1, CH	6.04, m	130.9, CH	6.02, m
12	132.6, CH	5.58, m	132.6, CH	5.57 ddd (14.5, 7.7, 7.1)	133.0, CH	5.58, m
13	34.5, CH_2	2.00, dd (14.3, 7.2)	34.5, CH_2	2.01,m	34.0, CH_2	2.26, dd (15.3, 7.0)
14	22.5, CH_2	1.36, sex (7.2)	22.5, C	1.36, sex (7.4)	22.5, CH_2	1.40, sex (7.4)
15	14.0, CH_3	0.87, t (7.2)	14.0, CH_3	0.86, t (7.4)	13.8, CH_3	0.89, t (7.4)
1′	75.0, CH	4.62, d (9.6)	74.9, CH	4.60, d (9.8)		
2′	72.1, CH	3.74, m	71.5, CH	3.83, t (9.2)		
3′	79.1, CH	3.22, m	79.0, CH	3.21, t (8.7)		
4′	70.3, CH	3.22, m	70.6, CH	3.18, t (8.7)		
5′	81.5, CH	3.20, m	78.4, CH	3.36 (under water peak)		
6′	61.2, CH_2	3.50, dd (11.0, 5.5) 3.65, dd (11.0, 5.2)	64.8, CH_2	4.32 d (11.6) 3.98 dd (11.6, 3.9)		
CO (OAc)			170.9 CO	-		
CH_3 (OAc)			21.2, CH_3	2.00, s		
OH-3		8.67, s		8.74, brs		
OH-3′				4.95, br		
OH-4′		4.90, dd (10.7, 2.9)		5.15, br		
OH-6′		4.59, d (5.5)				

Table S3. ^{1}H and ^{13}C NMR data of **6a** and **6b** ($CDCl_3$, 300 and 75 MHz).

	6a		6b	
Position	δ_C, type	δ_H, (*J* in Hz)	δ_C, type	δ_H, (*J* in Hz)
1	160.5, C		160.6, C	
2	110.0, CH	6.77, d (8.4)	109.7, CH	6.72, d (8.3)
3	138.2, CH	7.53, d (8.4)	138.7, CH	7.41, d (8.3
4	116.2, C-4		115.1, C	
5	119.1, CH	7.22, d (0.7)	119.2, CH	7.26, s
6	138.6, C		138.5, C	
7	149.6, C		149.6, C	
8	121.0, C		121.1, C	
9	109.3, C-9		109.2, C	
10	153.1, C		153.2, C	
11	152.0, C		152.0, C	
12	116.9, C-12		116.9, C	
13	184.4, CO		184.4, CO	
14	31.9, CH_2	2.68, dd (14.1, 10.6)<br>3.19, dd (14.0, 2.0)	29.7, CH_2	2.88, dd (14.2, 10.4)<br>3.34, dd (14.2, 3.0)
15	77.8, CH	3.73, ddd (10.4, 3.9, 2.1)	78.6, CH	5.18, dd (10.4, 3.2)
16	72.9, C		72.4, C	
17	26.6, CH_3	1.41, s	26.9, CH_3	1.34, s
18	23.6, CH_3	1.35, s	25.3, CH_3	1.38, s
19	64.6, CH_2	4.34, dd (10.9, 3.0)<br>4.43, dd (10.8, 3.5)	64.5, CH_2	4.35, dd (11.0, 3.0)<br>4.43, ddd (11.0, 3.4, 1.2)
20	44.9, CH	2.71, brs	44.9, CH	2.73, brd (2.6)
21	142.5, C		142.6, C	
22	112.3, CH_2	4.79, s<br>4.56, brd (0.6)	112.2, CH_2	4.57, brd (0.6)<br>4.80, brs
23	22.6, CH_3	1.84, s	22.6, CH_3	1.85, s
24	17.4, CH_3	2.34, d (0.7)	17.4, CH_3	2.36, d (0.7)
25	63.2, CH	5.38, m	63.2, CH	5.41, m
CO (OAc)-15			170.3, CO	
CH_3 (OAc)			20.8, CH_3	1.87,s
OH-1		12.61, s		12.65, s
OH-15		2.20, d (2.4)		
OH-25		5.00, d (3.8)		4.98, d (3.8)
